# Hypertension Diagnosis, Treatment, and Control in India

**DOI:** 10.1001/jamanetworkopen.2023.39098

**Published:** 2023-10-23

**Authors:** Jithin Sam Varghese, Nikhil Srinivasapura Venkateshmurthy, Nikkil Sudharsanan, Panniyammakal Jeemon, Shivani A. Patel, Harsha Thirumurthy, Ambuj Roy, Nikhil Tandon, K. M. Venkat Narayan, Dorairaj Prabhakaran, Mohammed K. Ali

**Affiliations:** 1 Emory Global Diabetes Research Center, Woodruff Health Sciences Center, Emory University, Atlanta, Georgia; 2 Hubert Department of Global Health, Rollins School of Public Health, Emory University, Atlanta, Georgia; 3 Public Health Foundation of India, New Delhi, India; 4 Professorship of Behavioral Science for Disease Prevention and Health Care, Technical University of Munich, Munich, Germany; 5 Heidelberg Institute of Global Health, Heidelberg University, Heidelberg, Germany; 6 Achutha Menon Centre for Health Science Studies, Sree Chitra Tirunal Institute for Medical Sciences and Technology, Trivandrum, India; 7 Leonard Davis Institute of Health Economics and Perelman School of Medicine, University of Pennsylvania, Philadelphia; 8 Department of Cardiology, All India Institute of Medical Sciences, New Delhi, India; 9 Department of Endocrinology and Metabolism, All India Institute of Medical Sciences, New Delhi, India; 10 Center for Chronic Disease Control, New Delhi, India; 11 Department of Family and Preventive Medicine, School of Medicine, Emory University, Atlanta, Georgia

## Abstract

**Importance:**

Hypertension is a major cause of morbidity and mortality worldwide. Previous efforts to characterize gaps in the hypertension care continuum—including diagnosis, treatment, and control—in India did not assess district-level variation. Local data are critical for planning, implementation, and monitoring efforts to curb the burden of hypertension.

**Objective:**

To examine the hypertension care continuum in India among individuals aged 18 to 98 years.

**Design, Setting, and Participants:**

The nationally representative Fifth National Family Health Survey study was conducted in 2 phases from June 17, 2019, to March 21, 2020, and from November 21, 2020, to April 30, 2021, among 1 895 297 individuals in 28 states, 8 union territories, and 707 districts of India.

**Exposures:**

District and state of residence, urban classification, age (18-39, 40-64, and ≥65 years), sex, and household wealth quintile.

**Main Outcomes and Measures:**

Hypertension was defined as a self-reported diagnosis or a newly measured blood pressure of 140/90 mm Hg or more. The proportion of individuals diagnosed (self-reported), the proportion of individuals treated among those diagnosed (self-reported medication use), and the proportion of individuals with blood pressure control among those treated (blood pressure <140/90 mm Hg [aged 18-79 years] or <150/90 mm Hg [aged ≥80 years]) were calculated based on national guidelines. Age-standardized estimates of treatment and control were also provided among the total with hypertension. To assess differences in the care continuum between or within states (ie, between districts), the variance was partitioned using generalized linear mixed models.

**Results:**

Of the 1 691 036 adult respondents (52.6% women; mean [SD] age, 41.6 [16.5] years), 28.1% (95% CI, 27.9%-28.3%) had hypertension, of whom 36.9% (95% CI, 36.4%-37.3%) received a diagnosis. Among those who received a diagnosis, 44.7% (95% CI, 44.1%-45.3%) reported taking medication (corresponding to 17.7% [95% CI, 17.5%-17.9%] of the total with hypertension). Among those treated, 52.5% (95% CI, 51.7%-53.4%) had blood pressure control (corresponding to 8.5% [95% CI, 8.3%-8.6%] of the total with hypertension). There were substantial variations across districts in blood pressure diagnosis (range, 6.3%-77.5%), treatment (range, 8.7%-97.1%), and control (range, 2.7%-76.6%). Large proportions of the variation in hypertension diagnosis (94.7%), treatment (93.6%), and control (97.3%) were within states, not just between states.

**Conclusions and Relevance:**

In this cross-sectional survey study of Indian adults, more than 1 in 4 people had hypertension, and of these, only 1 in 3 received a diagnosis, less than 1 in 5 were treated, and only 1 in 12 had blood pressure control. National mean values hide considerable state-level and district-level variation in the care continuum, suggesting the need for targeted, decentralized solutions to improve the hypertension care continuum in India.

## Key Points

**Question:**

What are the gaps in the hypertension care continuum (diagnosis, treatment, and control), and how do they vary by state, district, and sociodemographic groups in India?

**Findings:**

In this 2019-2021 national survey study of 1.7 million respondents, 28.1% had hypertension, of whom 36.9% received a diagnosis; 44.7% of those who received a diagnosis (17.7% of the total with hypertension) reported taking medication, and 52.5% of those treated (8.5% of the total with hypertension) achieved blood pressure control. Most of the variability in the hypertension care continuum was observed within, not between, states.

**Meaning:**

This study suggests that differences between states and between districts in states indicate the need for targeted, decentralized solutions to improve the hypertension care continuum in India.

## Teaser

This nationally representative survey study examines the hypertension care continuum—including diagnosis, treatment, and control—in India among individuals aged 18 to 98 years.

## Introduction

Hypertension is associated with 12.8% of all deaths globally.^1^ Many countries have implemented large-scale programs to diagnose and manage hypertension and other chronic diseases, with varying success.^2,3^ Of more than 1.3 billion people with hypertension globally, 82% live in low- and middle-income countries, and India alone is home to an estimated 220 million adults with hypertension.^4,5,6^ To address the burden of noncommunicable diseases, India launched the National Programme for Prevention and Control of Cancer, Diabetes, Cardiovascular Diseases and Stroke in 2010 (now known as the National Programme for Prevention and Control of Non-Communicable Diseases) under the National Health Mission for 100 districts across 21 states.^7^ However, few data are currently available to assess the success of and opportunities for improved control of high blood pressure at subnational levels.^8^

Previous efforts to examine the hypertension care continuum were limited to national and state levels, or were exclusively among older or younger adults, but not by sociodemographic groups within states or at district levels.^9,10,11,12^ Newer regional data may therefore strengthen “planning, implementation, and monitoring of investments” at the district level to improve health infrastructure and outreach services for hypertension—key objectives of the government of India’s national programs.^13,14,15^

Our primary objective was to describe the national, state-level, and district-level hypertension care continuum (prevalence, diagnosis, treatment, and control) in India, the world’s most populous country. Our secondary objective was to represent these data through a publicly available dashboard for stakeholders to help identify priorities for reducing the burden of hypertension in India and tracking the progress of national initiatives.

## Methods

### Study Population

The Fifth National Family Health Survey (NFHS) is a nationally representative survey conducted in 2 phases from June 17, 2019, to March 21, 2020, and from November 21, 2020, to April 30, 2021, in 707 districts from 28 states and 8 union territories and powered to provide estimates at the district level.^16^ Using a multistage stratified approach, 636 699 households within primary sampling units were randomly sampled from a list of households in which eligible participants (female participants, aged 15-49 years; male participants, aged 15-54 years) resided.^17^ Household and individual characteristics were collected using standardized instruments after obtaining written informed consent. The survey additionally collected data on blood pressure among all adults (aged ≥18 years) who were living in the same household as eligible participants. The overall approached sample consisted of 1 895 297 adults aged 18 to 98 years. This secondary analysis of publicly available deidentified data was exempted from ethical approval by the institutional review board of Emory University. This survey study followed the American Association for Public Opinion Research (AAPOR) reporting guideline and the Strengthening the Reporting of Observational Studies in Epidemiology (STROBE) reporting guideline.

We restricted our analysis to nonpregnant female participants and male participants who had a valid measurement of blood pressure (eFigure 1 in Supplement 1). The analytic sample consisted of 1 691 036 adults aged 18 to 98 years, representing a response rate of 89.2%. The analytic sample was similar to the excluded sample (eTable 1 in Supplement 1). Additional information on sampling and data collected are provided in the eMethods in Supplement 1.

### Data Collection

Trained examiners measured systolic and diastolic blood pressure 3 times at 5-minute intervals using standardized protocols and validated, self-calibrating electronic blood pressure monitors (OMRON Healthcare Inc) after a 5-minute period when the participant was asked to sit comfortably.^17^ The respondent was also asked to avoid eating, smoking, and exercising for 30 minutes before the measurement. The cuff size of the blood pressure monitor was based on the circumference of the bare upper arm measured using a Gulick tape measure. Blood pressure was measured on the left arm, positioned so that it was at heart level with the cuff placed over bare skin or over thin clothes. Consistent with 2016 Indian Council of Medical Research (ICMR) guidelines, we took the lowest of the first 2 measurements if their difference in systolic blood pressure was less than or equal to 5 mm Hg and the lowest of the 3 measurements otherwise.^18^ The blood pressure measurements in the households selected were high for both women (urban, 88.3%; rural, 93.0%) and men (urban, 79.3%; rural, 85.7%). Details on the interexaminer and intraexaminer reliability of blood pressure measurement are unavailable.^16,17^

Participants were also asked the question: “Before this survey, were you ever told you had high blood pressure by a doctor, nurse, or health practitioner on 2 or more occasions?” Medication status was asked only to those who self-reported a diagnosis of hypertension.

### Hypertension Care Continuum: Diagnosis, Treatment, and Control

We defined hypertension as self-reported or, among those without a prior diagnosis, as measured blood pressure of 140/90 mm Hg or more.^18^ We defined the hypertension care continuum using the following metrics: proportion of individuals who self-reported a diagnosis (ie, self-reported diagnosed hypertension prior to the survey among the total with hypertension) and proportion of those individuals treated (ie, self-reporting medication use). We defined the proportion of individuals with blood pressure control among those treated (<140/90 mm Hg for those aged 18-79 years and <150/90 mm Hg for those aged 80 years or older) based on ICMR guidelines for the management of hypertension.^18^ We also provided age-standardized estimates of treatment and control among all of those with hypertension. The definitions are summarized in eTable 2 in Supplement 1.

### Sociodemographic Variables

We estimated care continuum metrics by 3 individual-level sociodemographic factors: sex (male or female), age (18-39, 40-64, or ≥65 years), and schooling (none or missing, primary [up to 4th grade], secondary [up to 10th grade], or postsecondary). We also stratified by 2 household sociodemographic factors: rural residence (vs urban) and regional wealth quintile (urban and rural) from the household wealth index as provided by the NFHS.^19^

### Statistical Analysis

We report survey-weighted estimates accounting for the complex survey design and cluster-robust 95% CIs.^16^ Individual and household characteristics of the analytic sample were assessed by strata of residence (urban or rural) and sex.

Continuum performance indicators were estimated for the national sample, for states stratified by sociodemographic factors (residence, sex, age category, schooling, and regional wealth quintile), and for districts. Age-standardized estimates of the continuum indicators were computed for different strata at the national level based on the distribution of the total sample because different strata of schooling and wealth have different age distributions. We also calculated weighted estimates at the state and district levels that were not age standardized but would be relevant for local decision-making. We compared the estimates with those obtained when taking the mean of the last 2 blood pressure measurements as a sensitivity analysis.

To assess whether the differences in the care continuum were greater between or within states (ie, between districts), we partitioned the variance in the care continuum at both levels using variance partition coefficients from generalized linear mixed models with state-level and district-level intercepts, adjusting for sociodemographic variables.^20^ To illustrate the variability between and within states, we present examples of 2 states from regions with moderate to high burdens of hypertension, Karnataka from South India and Meghalaya from North East India.

To further aid policy and priority decision-making, we developed a dashboard^21^ to visually depict the disparities in the hypertension care continuum using Shiny by RStudio. All analyses and visualization were carried out using R, version 4.2.0 (R Project for Statistical Computing) using srvyr, version 1.1.1 and other packages.^22,23,24,25^

## Results

The analytic sample of 1 691 036 adult respondents was 52.6% women and 47.4% men, with a mean (SD) age of 41.6 (16.5) years (Table 1). Nationally, more than three-fourths of the population lived in rural areas from 2019 to 2021. More than half the respondents were younger than 40 years of age, and almost 90% were aged 18 to 64 years. The mean (SD) systolic blood pressure and the mean (SD) diastolic blood pressure were 120.4 (18.3) mm Hg and 79.6 (10.5) mm Hg, respectively, for women and 124.7 (15.9) mm Hg and 81.6 (10.4) mm Hg, respectively, for men.

**Table 1.** Characteristics of Participants in the Analytic Sample for Estimating Care Cascade of Hypertension in India (N = 1 691 036)

Characteristic	Participants, % (95% CI)^a^					
	Total		Urban		Rural	
	Women (n = 889 468)	Men (n = 801 568)	Women (n = 218 587)	Men (n = 198 903)	Women (n = 670 881)	Men (n = 602 665)
Age category, y						
18-39	50.1 (49.9-50.2)	49.1 (48.9-49.3)	49.5 (49.1-49.9)	49.6 (49.3-50.0)	50.3 (50.2-50.5)	48.8 (48.6-49.1)
40-64	39.4 (39.2-39.5)	38.7 (38.6-38.9)	40.3 (40.0-40.5)	39.2 (38.9-39.5)	38.9 (38.8-39.1)	38.5 (38.3-38.7)
≥65	10.6 (10.5-10.7)	12.2 (12.1-12.3)	10.2 (10.0-10.5)	11.1 (10.9-11.4)	10.7 (10.6-10.9)	12.7 (12.5-12.8)
Educational level						
None	37.1 (36.8-37.4)	17.4 (17.2-17.6)	22.3 (21.8-22.8)	9.5 (9.2-9.8)	44.0 (43.7-44.3)	21.2 (21.0-21.5)
Primary (≤4th grade)	13.8 (13.7-14.0)	15.1 (14.9-15.2)	12.6 (12.3-12.8)	11.4 (11.1-11.7)	14.4 (14.3-14.6)	16.8 (16.7-17.0)
Secondary (5th-10th grade)	36.7 (36.5-36.9)	49.5 (49.3-49.7)	43.3 (42.9-43.7)	50.8 (50.3-51.3)	33.5 (33.3-33.8)	48.9 (48.6-49.2)
Postsecondary (≥11th grade)	12.4 (12.2-12.6)	18.0 (17.7-18.3)	21.8 (21.3-22.3)	28.3 (27.7-28.9)	8.0 (7.9-8.2)	13.0 (12.7-13.4)
Household wealth quintile (by residence)						
Lowest	18.6 (18.3-18.9)	18.0 (17.6-18.3)	19.4 (18.6-20.1)	19.4 (18.6-20.1)	18.2 (17.9-18.6)	17.3 (17.0-17.6)
Low	19.6 (19.4-19.8)	19.3 (19.1-19.6)	20.2 (19.7-20.7)	20.2 (19.7-20.7)	19.3 (19.1-19.5)	18.9 (18.6-19.2)
Medium	20.3 (20.1-20.5)	20.4 (20.2-20.6)	20.3 (19.9-20.8)	20.3 (19.9-20.8)	20.3 (20.1-20.5)	20.4 (20.2-20.7)
High	20.7 (20.4-20.9)	21.2 (20.9-21.4)	20.3 (19.8-20.8)	20.3 (19.8-20.8)	20.9 (20.6-21.1)	21.6 (21.3-21.9)
Highest	20.8 (20.5-21.2)	21.1 (20.8-21.5)	19.9 (19.2-20.6)	19.7 (19.0-20.5)	21.3 (20.9-21.7)	21.8 (21.5-22.2)
Mean blood pressure measurement, mm Hg						
Systolic	120.4 (120.3-120.5)	124.7 (124.6-124.8)	120.6 (120.5-120.8)	125.4 (125.3-125.6)	120.3 (120.2-120.3)	124.3 (124.2-124.4)
Diastolic	79.6 (79.6-79.7)	81.6 (81.5-81.6)	79.9 (79.8-80.0)	82.1 (82.0-82.2)	79.5 (79.5-79.6)	81.4 (81.3-81.4)
Hypertension						
Self-reported or high blood pressure	27.1 (26.9-27.3)	28.4 (28.1-28.6)	29.1 (28.7-29.4)	31.0 (30.6-31.5)	26.2 (26.0-26.4)	27.1 (26.9-27.3)
Self-reported	12.6 (12.5-12.8)	9.1 (9.0-9.3)	15.0 (14.6-15.3)	11.2 (10.9-11.5)	11.6 (11.4-11.8)	8.1 (8.0-8.3)
Blood pressure category, mm Hg						
<120/80	40.2 (40.0-40.4)	27.3 (27.1-27.6)	38.3 (37.9-38.7)	24.7 (24.3-25.2)	41.0 (40.8-41.3)	28.6 (28.3-28.9)
120/80 to <140/90	39.7 (39.5-39.9)	48.8 (48.5-49.0)	40.6 (40.2-41.0)	49.5 (49.1-50.0)	39.3 (39.1-39.5)	48.4 (48.1-48.6)
140/90 to <160/100	14.4 (14.3-14.6)	17.7 (17.6-17.9)	15.4 (15.1-15.7)	19.2 (18.9-19.5)	14.0 (13.8-14.1)	17.0 (16.9-17.2)
≥160/100 mm Hg	5.7 (5.6-5.8)	6.2 (6.1-6.3)	5.6 (5.5-5.8)	6.5 (6.3-6.7)	5.7 (5.6-5.8)	6.0 (5.9-6.1)

^a^ All values are percentages (95% CIs) accounting for survey design. Estimates are not age standardized. The Household Wealth Index, as provided by the Demographic and Health Surveys, was computed as the first principal component from survey responses regarding possession of assets and quality of housing assessed separately for urban and rural areas.

### National-Level Care Continuum

The age-standardized prevalence of hypertension nationally was 28.1% (95% CI, 27.9%-28.3%) and was higher in urban areas (32.6% [95% CI, 32.2%-33.0%]) relative to rural areas (25.9% [95% CI, 25.7%-26.1%]) (Table 2). The prevalence was higher among men (30.6% [95% CI, 30.3%-30.8%]) relative to women (25.7% [95% CI, 25.5%-25.9%]), and it was higher among participants at older ages (≥65 years, 54.3% [95% CI, 53.8%-54.8%]; 18-39 years, 14.9% [95% CI, 14.8%-15.1%]) and among those with greater household wealth (highest, 31.1% [95% CI, 30.7%-31.4%]; lowest, 25.4% [95% CI, 25.1%-25.8%]) compared with their respective counterparts. A higher prevalence of hypertension among men, older individuals, and wealthier individuals was observed in both urban and rural areas. The prevalence of hypertension did not vary by educational level at the national level. Crude estimates of the care continuum are provided in eTable 3 in Supplement 1.

**Table 2.** Sociodemographic Variations in Care Continuum in India (N = 1 691 036)

Characteristic	Participants, % (95% CI)^a^											
	Total				Urban				Rural			
	Hypertension	Diagnosed^b^	Treated^c^	Controlled^d^	Hypertension	Diagnosed^b^	Treated^c^	Controlled^d^	Hypertension	Diagnosed^b^	Treated^c^	Controlled^d^
Total	28.1 (27.9-28.3)	36.9 (36.4-37.3)	44.7 (44.1-45.3)	52.5 (51.7-53.4)	32.6 (32.2-33.0)	39.9 (39.1-40.8)	56.3 (54.9-57.6)	50.4 (49.0-51.9)	25.9 (25.7-26.1)	35.4 (34.8-35.9)	38.8 (38.0-39.6)	53.9 (52.9-55.0)
Sex												
Women	25.7 (25.5-25.9)	44.6 (44.0-45.1)	42.2 (41.5-42.9)	55.6 (54.6-56.6)	30.1 (29.7-30.5)	47.9 (46.8-48.9)	54.0 (52.5-55.5)	53.2 (51.4-55.0)	23.7 (23.5-23.9)	43.1 (42.4-43.8)	36.9 (36.0-37.8)	57.1 (55.8-58.4)
Men	30.6 (30.3-30.8)	28.4 (27.9-28.8)	49.3 (48.5-50.1)	47.4 (46.0-48.7)	35.1 (34.6-35.6)	32.2 (31.4-33.0)	59.9 (58.3-61.5)	46.4 (44.1-48.7)	28.2 (27.9-28.5)	26.3 (25.8-26.8)	42.8 (41.8-43.9)	48.1 (46.5-49.8)
Age category, y												
18-39	14.9 (14.8-15.1)	31.5 (30.8-32.2)	23.8 (22.9-24.7)	61.3 (59.7-62.9)	15.6 (15.2-15.9)	28.6 (27.2-29.9)	27.2 (25.3-29.0)	57.6 (54.6-60.5)	14.7 (14.5-14.9)	32.6 (31.8-33.4)	22.7 (21.7-23.7)	63.4 (61.6-65.2)
40-64	37.2 (36.9-37.5)	39.5 (39.1-40.0)	61.8 (61.1-62.4)	43.7 (43.1-44.4)	40.2 (39.6-40.7)	44.5 (43.7-45.3)	70.0 (68.8-71.2)	44.6 (43.5-45.7)	35.4 (35.1-35.7)	36.6 (36.1-37.1)	56.0 (55.2-56.8)	43.0 (42.2-43.8)
≥65	54.3 (53.8-54.8)	51.3 (50.7-51.9)	77.1 (76.5-77.8)	44.4 (43.6-45.2)	60.1 (59.1-61.1)	59.8 (58.6-61.0)	83.9 (82.8-85.0)	45.8 (44.4-47.2)	50.4 (49.9-50.9)	45.7 (45.1-46.4)	71.7 (70.8-72.5)	43.3 (42.4-44.2)
Educational level												
None	27.6 (27.4-27.9)	36.3 (35.6-37.0)	41.6 (40.7-42.6)	47.4 (46.2-48.6)	32.8 (32.1-33.5)	39.0 (37.4-40.6)	52.1 (49.4-54.7)	44.5 (42.1-47.0)	26.5 (26.2-26.8)	35.6 (34.8-36.4)	38.8 (37.7-40.0)	48.5 (47.1-49.8)
Primary (≤4th grade)	28.8 (28.5-29.1)	36.0 (35.3-36.7)	46.8 (45.6-48.0)	50.8 (49.1-52.5)	34.8 (34.0-35.5)	38.3 (36.9-39.6)	58.9 (56.5-61.3)	46.3 (43.6-49.0)	26.7 (26.4-27.0)	35.1 (34.3-36.0)	42.0 (40.6-43.5)	53.2 (51.0-55.3)
Secondary (5th-10th grade)	28.1 (27.9-28.3)	37.0 (36.5-37.5)	45.8 (45.1-46.5)	53.2 (51.9-54.5)	32.9 (32.5-33.4)	40.0 (39.1-40.9)	57.9 (56.4-59.4)	50.1 (48.0-52.1)	25.3 (25.1-25.6)	35.2 (34.5-35.8)	37.9 (36.9-38.8)	55.6 (53.8-57.3)
Postsecondary (≥11th grade)	28.2 (27.8-28.7)	39.4 (38.6-40.3)	46.9 (45.7-48.0)	59.6 (57.1-62.2)	31.0 (30.3-31.7)	42.1 (40.8-43.3)	55.4 (53.5-57.3)	57.1 (53.6-60.5)	24.4 (23.8-25.0)	35.8 (34.7-36.9)	34.6 (33.0-36.2)	63.6 (60.0-67.2)
Household wealth quintile^e^												
Lowest	25.4 (25.1-25.8)	32.0 (31.1-32.9)	37.2 (36.0-38.4)	54.7 (52.6-56.7)	29.0 (28.3-29.8)	34.9 (33.1-36.6)	45.1 (42.5-47.7)	49.9 (46.8-53.1)	23.6 (23.2-23.9)	30.4 (29.4-31.4)	32.2 (30.6-33.7)	58.4 (55.7-61.0)
Low	26.7 (26.4-27.0)	35.9 (35.1-36.6)	41.9 (40.9-42.9)	51.5 (49.7-53.3)	32.0 (31.3-32.6)	38.2 (36.9-39.5)	55.3 (53.2-57.5)	47.3 (44.5-50.0)	23.9 (23.6-24.2)	34.5 (33.6-35.5)	34.0 (32.7-35.3)	55.2 (53.0-57.5)
Medium	27.9 (27.6-28.2)	36.8 (36.1-37.5)	44.5 (43.4-45.5)	52.3 (50.5-54.1)	33.3 (32.6-33.9)	39.7 (38.5-40.9)	59.6 (57.6-61.7)	50.4 (47.5-53.2)	25.1 (24.8-25.5)	35.4 (34.4-36.3)	36.5 (35.2-37.8)	53.8 (51.5-56.2)
High	29.0 (28.7-29.3)	37.3 (36.5-38.0)	47.2 (46.2-48.3)	53.2 (51.5-54.9)	33.9 (33.2-34.6)	41.7 (40.4-43.0)	59.3 (57.2-61.4)	51.7 (48.7-54.7)	26.6 (26.3-27.0)	35.2 (34.3-36.1)	41.4 (40.0-42.7)	54.1 (52.0-56.2)
Highest	31.1 (30.7-31.4)	40.7 (39.9-41.4)	48.1 (47.0-49.2)	51.9 (50.2-53.5)	34.8 (34.0-35.5)	44.3 (42.8-45.8)	58.7 (56.3-61.0)	51.7 (48.4-55.0)	29.4 (29.1-29.8)	39.2 (38.3-40.1)	43.8 (42.4-45.1)	52.0 (50.1-53.9)

^a^ Estimates (95% CIs) are standardized to age distribution in overall sample.

^b^ Among those with self-reported hypertension or high blood pressure (≥140/90 mm Hg).

^c^ Among those with self-reported hypertension (ie, diagnosed).

^d^ Among those taking medication for hypertension (ie, treated).

^e^ We used the Household Wealth Index computed as the first principal component from survey responses regarding possession of assets and quality of housing assessed separately for urban and rural areas, as provided by Demographic and Health Survey.

**Figure 1.**
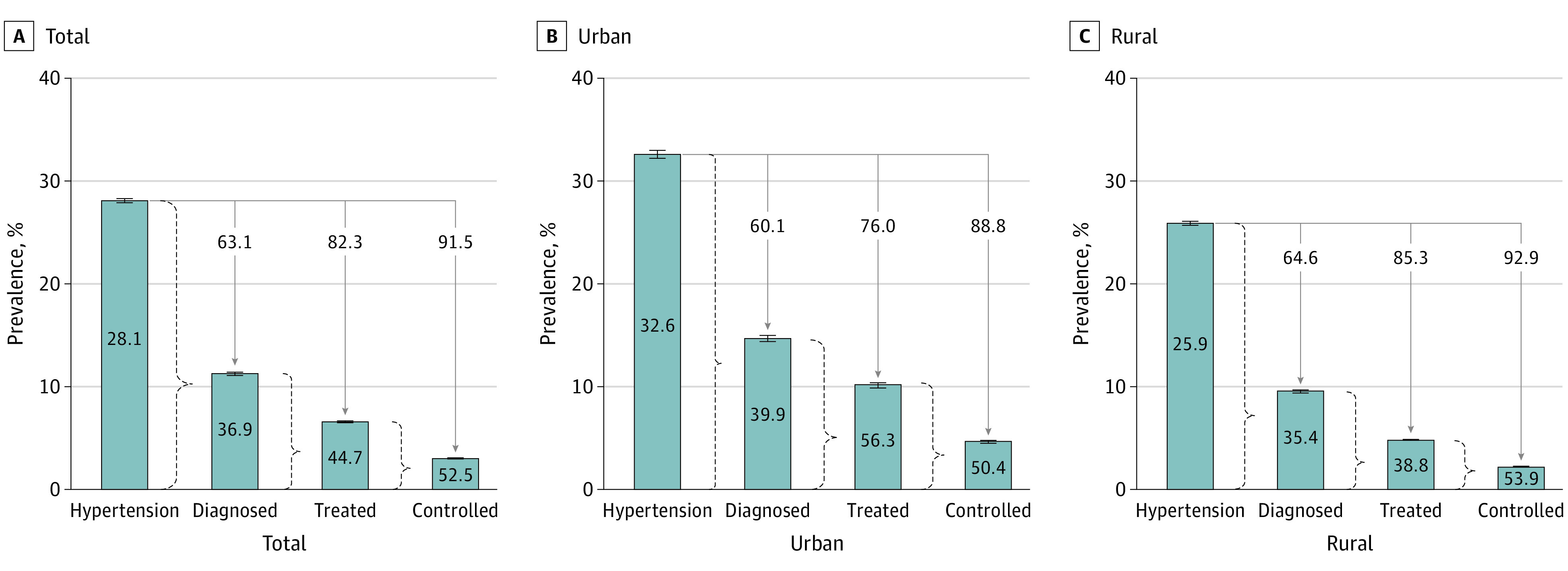
National-Level Care Continuum for Indian Adults by Residence (N = 1 691 036) All columns are survey-weighted percentages in total population. We performed age standardization to the distribution of the within-sample total population separately for total population, population with hypertension, diagnosed population, and treated population. This procedure harmonizes the age distribution within each category (total, hypertension, diagnosed, and treated). The values should therefore not be sequentially multiplied to determine prevalence within total population. The values inside the bars are proportions of diagnosed hypertension among patients with hypertension, treated among diagnosed hypertension, and controlled among treated hypertension (from Table 2). Values above the bars are relative to all patients with hypertension (100 − % diagnosed among hypertension, 100 − % treated among hypertension, and 100 − % controlled among hypertension).

Among all adults with hypertension, 36.9% (95% CI, 36.4%-37.3%) reported receiving a diagnosis (Figure 1). Diagnosed hypertension was higher in urban (39.9% [95% CI, 39.1%-40.8%]) compared with rural areas (35.4% [95% CI, 34.8%-35.9%]), higher among older age groups (≥65 years, 51.3% [95% CI, 50.7%-51.9%]; 18-39 years, 31.5% [95% CI, 30.8%-32.2%]), those with greater household wealth (highest, 40.7% [95% CI, 39.9%-41.4%]; lowest, 32.0% [95% CI, 31.1%-32.9%]), and those with a higher level of schooling (postsecondary, 39.4% [95% CI, 38.6%-40.3%]; none, 36.3% [95% CI, 35.6%-37.0%]).

Of adults with diagnosed hypertension, 44.7% (95% CI, 44.1%-45.3%) reported taking medication, corresponding to 17.7% (95% CI, 17.5%-17.9%) of those with hypertension (Figure 1; eTable 4 in Supplement 1). Among those diagnosed, 56.3% (95% CI, 54.9%-57.6%) reported using medication in urban areas, and 38.8% (95% CI, 38.0%-39.6%) reported using medication in rural areas. These estimates correspond to 24.0% (95% CI, 23.4%-24.5%) and 14.7% (95% CI, 14.4%-14.9%) of those with hypertension in urban and rural areas, respectively. The proportions of those who received a diagnosis and who were treated were higher among men (men, 49.3% [95% CI, 48.5%-50.1%]; women, 42.2% [95% CI, 41.5%-42.9%]), older participants (≥65 years, 77.1% [95% CI, 76.5%-77.8%]; 18-39 years, 23.8% [95% CI, 22.9%-24.7%]), and those with the greatest household wealth (lowest, 37.2% [95% CI, 36.0%-38.4%]; highest, 48.1% [95% CI, 47.0%-49.2%]), but the proportion did not increase with higher educational level. Among the adults who received a diagnosis, the distributions of those with treated and controlled hypertension, those with treated and uncontrolled hypertension, or those with untreated hypertension are presented in eFigure 2 in Supplement 1. Estimates of treated and controlled hypertension among those with hypertension by sociodemographic group are provided in eTable 4 in Supplement 1.

Among those who received a diagnosis and were treated with medication, 52.5% (95% CI, 51.7%-53.4%) had controlled blood pressure, corresponding to 8.5% (95% CI, 8.3%-8.6%) of all those with hypertension (Figure 1; eTable 4 in Supplement 1). Among treated adults, the proportion with controlled hypertension was 50.4% (95% CI, 49.0%-51.9%) in urban areas and 53.9% (95% CI, 52.9%-55.0%) in rural areas. These estimates correspond to 11.2% (95% CI, 10.8%-11.5%) and 7.1% (95% CI, 7.1%-7.3%) of those with hypertension in urban and rural areas, respectively. Controlled hypertension among those treated was higher among women (55.6% [95% CI, 54.6%-56.6%]) than men (47.4% [95% CI, 46.0%-48.7%]) and adults aged 18 to 39 years (61.3% [95% CI, 59.7%-62.9%]) compared with those aged 40 to 64 years (43.7% [95% CI, 43.1%-44.4%]) and those aged 65 years or older (44.4% [95% CI, 43.6%-45.2%]). Hypertension control was also higher among those with higher education (no higher education, 47.4% [95% CI, 46.2%-48.6%]; postsecondary education, 59.6% [95% CI, 57.1%-62.2%]) but did not differ by household wealth (lowest, 54.7% [95% CI, 52.6%-56.7%]; low, 51.5% [95% CI, 49.7%-53.3%]; medium, 52.3% [95% CI, 50.5%-54.1%]; highest, 51.9% [95% CI, 50.2%-53.5%]). Our results were similar when using the mean of the second and third measurements of blood pressure (eTable 5 in Supplement 1) instead of the lowest measurements (eTable 6 and eFigure 3 in Supplement 1).

### State-Level Care Continuum

**Figure 2.**
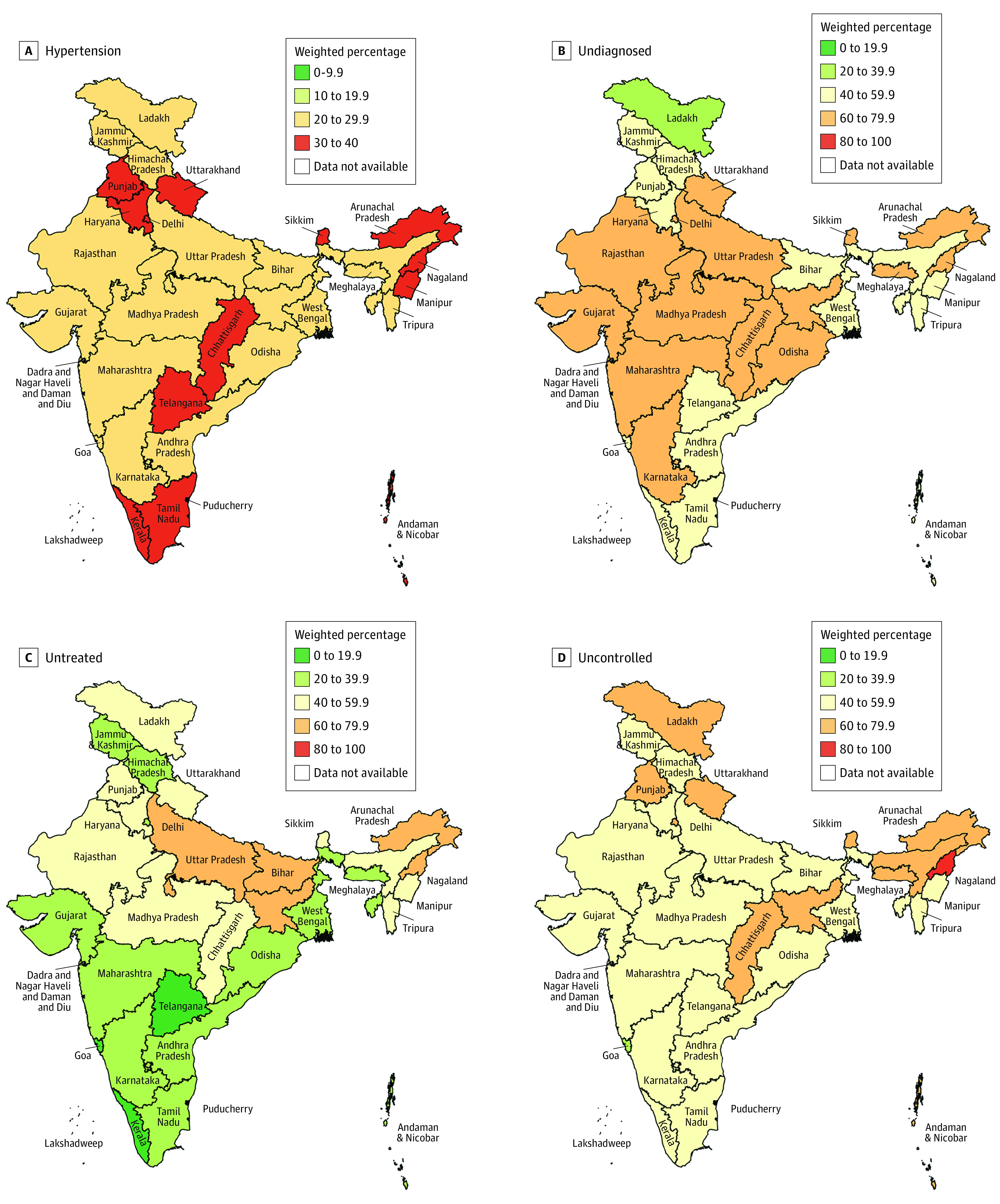
State-Level Unmet Need in Hypertension Care Continuum (N = 1 691 036) All values are survey-weighted percentages (not age standardized). Undiagnosed are among those with hypertension. Untreated and uncontrolled are among those diagnosed with hypertension and among those treated, respectively. We report weighted estimates at the state level that were not age standardized and relevant for local decision-making in this article.

The prevalence of hypertension was similar among the southern states (Kerala, Tamil Nadu, Karnataka, Telangana, and Andhra Pradesh), union territories (Andaman and Nicobar Islands, Lakshadweep, and Puducherry), and Goa compared with other parts of the country (Figure 2; median percentage of states: southern states, 29.9% [IQR, 29.1%-31.4%] vs rest of India, 26.8% [24.4%-32.0%]). A higher prevalence of hypertension was observed in urban vs rural areas for all states (eFigure 4 in Supplement 1).

The proportions of participants with diagnosed hypertension were similar between southern states and the rest of India (eFigure 5 in Supplement 1). However, the proportion of participants with treated hypertension and the proportion of participants with controlled hypertension were higher among the southern states. Disparities in diagnosis, treatment, and control between sociodemographic groups within each state beyond the state-level heterogeneity observed in Figure 2 are published on the interactive Hypertension Care Continuum dashboard.^21^

### District-Level Care Continuum

**Figure 3.**
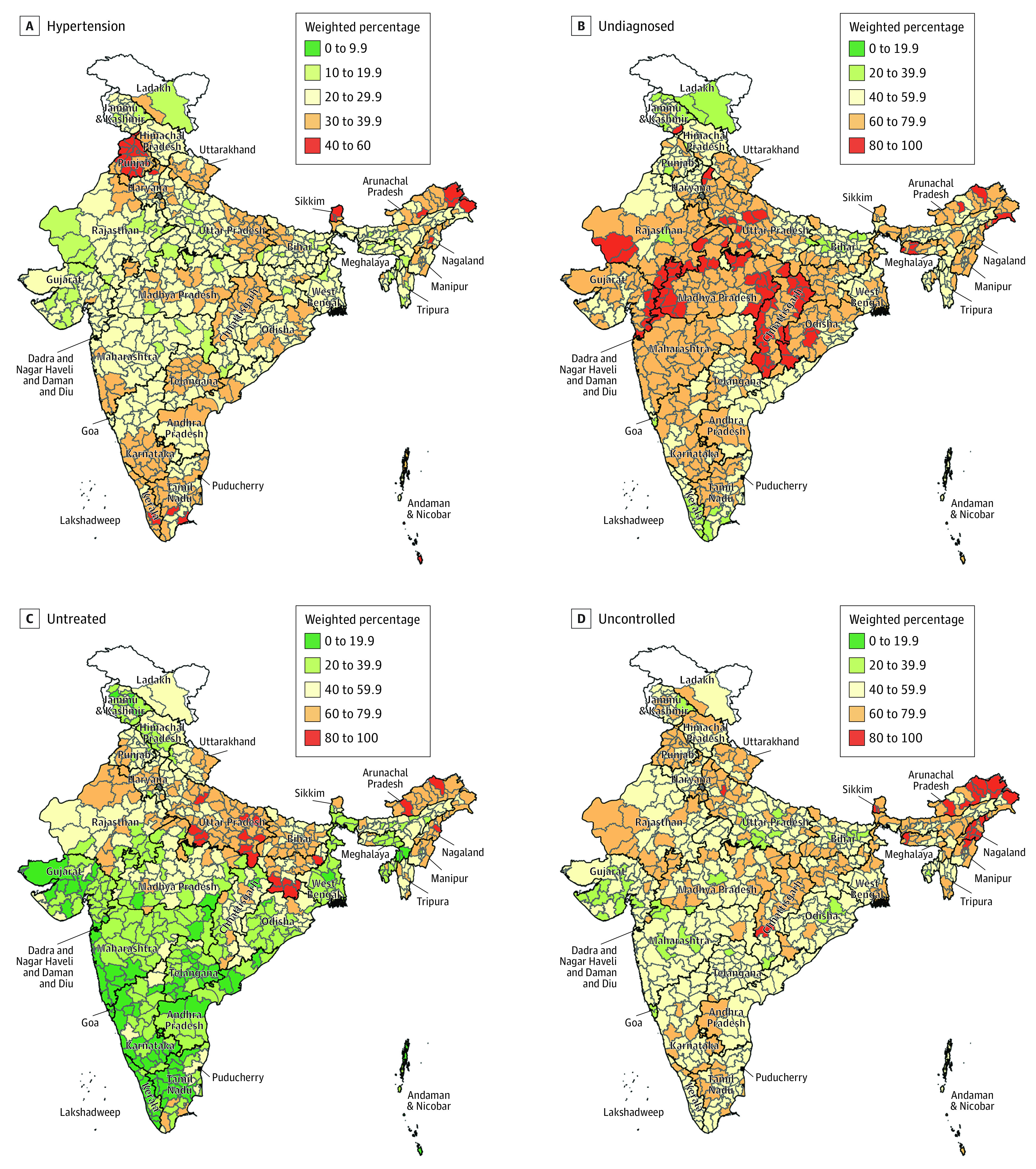
Care Continuum in Analytic Sample by Urban and Rural Residence for 707 Districts (N = 1 691 036) All values are survey-weighted percentages (not age standardized). Undiagnosed are among those with hypertension. Untreated and uncontrolled are among those diagnosed with hypertension and among those treated, respectively. We report weighted estimates at the district-level that were not age standardized and relevant for local decision-making in this article.

There was considerable within-state variation in the hypertension care continuum (Figure 3) such that 94.7% of the variance in the proportion of participants who received a diagnosis (range, 6.3%-77.5%), 93.6% of the variance in the proportion of participants treated among those who received a diagnosis (range, 8.7%-97.1%), and 97.3% of the variance in the proportion of participants with blood pressure control among those treated (range, 2.7%-76.6%) were at the district level or below, with the remaining at the state level (between states). We visualized this variability between and within states from all regions in eFigure 6 in Supplement 1.

We illustrated this variability in Meghalaya and Karnataka. In Meghalaya, the 5 districts of Garo Hills (median prevalence, 21.8% [range, 17.1%-23.7%]) had a similar prevalence as the 2 districts of Jaintia Hills (median prevalence, 19.8% [range, 18.6%-20.9%]) and 3 districts of Khasi Hills (median prevalence, 23.1% [range, 20.2%-24.8%]), although the proportions of those diagnosed were much lower in Garo Hills (median prevalence, 18.6% [range, 14.8%-23.8%]) than Jaintia Hills (median prevalence, 41.1% [range, 36.7%-45.5%]) and Khasi Hills (median prevalence, 29.4% [range, 27.7%-42.3%]) (eFigure 7A in Supplement 1).

In Karnataka, there was substantial between-district heterogeneity in treatment among those who received a diagnosis but less heterogeneity in blood pressure control among those treated between districts with similar prevalences. Chikmagalur (31.5% [95% CI, 28.8%-34.3%]), Udupi (34.0% [95% CI, 31.5%-36.6%]), Chitradurga (34.8% [95% CI, 32.0%-37.5%]), and Shimoga (34.0% [95% CI, 29.0%-39.0%]) had similar prevalences of hypertension. The proportions of participants treated were higher in Chikmagalur (81.3% [95% CI, 74.5%-88.0%]) and Udupi (91.3% [95% CI, 87.6%-95.1%]) compared with Chitradurga (61.6% [95% CI, 45.9%-77.2%]) and Shimoga (55.7% [95% CI, 38.1%-73.3%]) (eFigure 7B in Supplement 1). Similarly, the proportion of those with controlled hypertension (eFigure 7C in Supplement 1) was higher in Chikmagalur (42.5% [95% CI, 33.6%-51.4%]) and Udupi (44.4% [95% CI, 39.9%-48.8%]) compared with Chitradurga (39.6% [95% CI, 30.8%-48.4%]) and Shimoga (35.5% [95% CI, 29.2%-41.9%]).

## Discussion

Of the estimated 28% of adults older than 18 years with high blood pressure in India, nearly 2 in 3 remain without a diagnosis across all states and in both urban and rural areas.^26^ Among those with a diagnosis, only half were treated; treatment rates were higher in southern and western India and lower in other parts of the country. Among those treated, nearly half did not have their blood pressure under control. Cumulatively, more than 90% of adults with hypertension in India were either undiagnosed, untreated, or treated but with uncontrolled hypertension.

There was substantial variability across sociodemographic groups in the prevalence, diagnosis, treatment, and control of hypertension. Although the prevalence of hypertension was higher among men, the proportion of those receiving a diagnosis was higher among women.^11,27^ Women were less likely to be taking medication, but treated women were more likely than treated men to have controlled hypertension.^10,11^ In a study of 44 low- and middle-income countries as well as other studies from India, being female was associated with higher rates of diagnosis, treatment, and control of hypertension.^3,10^ However, the National Non-Communicable Disease Monitoring Survey (NNMS) from India observed sex differences in the diagnosis of hypertension but not treatment or control after adjusting for other characteristics.^11^ The observed sex differences in the hypertension care continuum is likely due to a combination of social and biological factors. A comparison of age-standardized and crude estimates also suggests differences in the age distribution at different stages of the care continuum. The proportion of individuals who received a diagnosis of hypertension did not vary with level of schooling,^11^ but the proportions of those with treated and controlled hypertension were higher among those with a higher level of schooling. The proportions of individuals who received a diagnosis and were treated were higher among older adults and and those with greater household wealth.^10,11^

The reasons for the greater differences in hypertension diagnosis, treatment, and control being between districts in a state and not between states are likely multifactorial, consisting of health system, geography (eg, altitude and temperature), and patient (eg, age, diet, and smoking) characteristics that differ between regions.^28,29^ Prior and recent data show that there are between-district differences in health-seeking behaviors across India.^16^ Furthermore, clinician (eg, type of clinician and practice variation) and system (eg, physical and financial access to clinics) factors also differ between states and districts.^30,31^

The high unmet need in hypertension diagnoses in India has been identified previously, although none of these studies provide comprehensive estimates for all age groups and district-level precision in estimates.^12,32^ In 2017 and 2018, the NNMS surveyed 10 659 adults aged 18 to 69 years from 26 states and estimated a hypertension prevalence of 28.5%.^11^ Among those with hypertension, 27.9% received a diagnosis, 14.5% were treated (52.0% among those who received a diagnosis), and 12.6% had blood pressure control (86.9% among those treated). The NFHS-4 (conducted from 2015 to 2016) and Longitudinal Ageing Study in India (LASI; conducted from 2017 to 2019) provided estimates for those aged 15 to 49 years (n = 731 864; prevalence, 18.1%; diagnosed, 44.7%; treated among those diagnosed, 29.8%; controlled among those treated, 59.4%) and those older than 45 years (n = 72 262; prevalence, 45.9%; diagnosed, 55.7%; treated among those diagnosed, 69.8%; controlled among those diagnosed, 56.9%), respectively.^9,10,33^ The NFHS-4 and LASI provided estimates by sociodemographic group and state.^14,15^

To improve the care continuum for hypertension in India, our data are consistent with previous studies that suggest diagnosis is a critical step in realizing the downstream indicators, such as treatment and control. Data from other low- and middle-income countries show that diagnosis is also the largest gap (only 4 in 10 individuals receive a diagnosis) relative to high-income countries (7 in 10 individuals receive a diagnosis).^3,6,34^ Screening and linkage to care are therefore critical, as evidenced by previous data.^35,36,37^ The current national guidelines recommend universal screening for those older than 30 years and emphasize the cost-effective nature of hypertension management as a public health intervention.^18^ Studies within India also offer opportunities to improve hypertension diagnosis by linking frontline health workers who carry out hypertension screening at the community level with physicians at the facility level through an information technology–enabled platform.^38^ Under the Ayushman Bharat Comprehensive Primary Health Care (CPHC) program for screening and referral for noncommunicable diseases, digitization of screening records by frontline workers can enable surveillance of hypertension burdens.^39^ Concerted strategies for hypertension treatment and control may offer models for India to emulate.^40,41,42^ Hypertension control can also be facilitated by providing physicians with the latest evidence-based guidelines on treating hypertension through decision support systems embedded within the CPHC Non-Communicable Disease software.^15,38^ Furthermore, population-based strategies, such as policy-mandated reductions in the salt content of packaged foods, food labeling, low sodium or salt substitutes,^43^ reducing particulate exposure, and improved built environments, can complement the clinical efforts.

### Strengths and Limitations

Our study has some strengths. To our knowledge, this study is among the largest of its kind, consisting of nearly 1.7 million respondents with high response rates and providing data at the district level and for sociodemographic groups. The study used validated protocols for blood pressure measurement, including cuff size selection, and our presentation offers easy-to-use visualization of results.

Our study also has some limitations. First, while the hypertension care continuum is an invaluable tool to visualize gaps at 1 time point, these data hide the dynamic nature of hypertension treatment and control, which argues for systems of ongoing surveillance.^44,45^ Second, hypertension among those who did not self-report a physician diagnosis was based on blood pressure measurements at a single time point.^46^ The ICMR guidelines for diagnosis of hypertension require a minimum of 2 sets of readings on 2 different occasions, which are at least 1 to 4 weeks apart.^18^ However, single blood pressure measurements have been used in surveillance studies globally, and this approach offers consistency and comparability across studies. Third, diagnosis and treatment were based on self-report and not validated through medical records. Although an individual’s awareness of their diagnosis is most directly relevant for care seeking, previous studies suggest that self-reported diagnosis of hypertension may have only moderate sensitivity (86%).^44,47^ Fourth, we did not have data on older adults living by themselves or institutionalized and noncivilian adults.^16^

## Conclusions

In India, nationally, more than 1 in 4 people have hypertension, and cumulatively, more than 90% of adults with hypertension are either undiagnosed, untreated, or treated but with uncontrolled hypertension. These summary data, however, hide district-level and sociodemographic differences. Thus, as our data indicate, the characterization and visualization of India’s hypertension care continuum nationally, at the state and district levels, and across sociodemographic groups present opportunities to tailor implementation of programs to prevent and control the burden of high blood pressure.

## Notes

Supplement 1. **eTable 1.** Characteristics of Participants in Analytic Sample Versus Those Excluded, N = 1,895,297 **eTable 2.** Definitions of Disease and Care Continuum of Hypertension **eTable 3.** Crude Estimates of Socio-Demographic Variations in Care Continuum in India Relative to Population With Hypertension, N = 1,691,036 **eTable 4.** Socio-Demographic Variations in Care Continuum in India Relative to Population With Hypertension, N = 1,691,036 **eTable 5.** Average Values of Systolic and Diastolic Blood Pressure for Each of the Three Measurements, N = 1,691,036 **eTable 6.** Socio-Demographic Disparities in Hypertension Care Continuum in India When Using Average of Last 2 Blood Pressure Measurements, N = 1,691,036 **eFigure 1.** Flowchart of Analytic Sample **eFigure 2.** Distribution of Diagnosed Hypertension by Treatment and Control Status **eFigure 3.** Distribution of Diagnosed Hypertension by Treatment and Control Status When Using Average of Last 2 Blood Pressure Measurements **eFigure 4.** State-Level Care Continuum **eFigure 5.** Heatmap for Care Continuum **eFigure 6.** Distribution of Between- and Within-State Variability in Hypertension Care Continua for Selected States **eFigure 7.** Disparities Within States at the District Level **eMethods.** National Family Health Survey-5, 2019-21: Survey Design and Data Collection

Supplement 2.
Data Sharing Statement
