# Supplementary material for: Hypertension Diagnosis, Treatment, and Control in India
Source: JAMA Netw Open. 2023 Oct 23;6(10):e2339098. doi: 10.1001/jamanetworkopen.2023.39098 (PMC10594142; doi:10.1001/jamanetworkopen.2023.39098)
Supplement: Supplement 1. — eTable 1. Characteristics of Participants in Analytic Sample Versus Those Excluded, N = 1,895,297 eTable 2. Definitions of Disease and Care Continuum of Hypertension eTable 3. Crude Estimates of Socio-Demographic Variations in Care Continuum in India Relative to Population With Hypertension, N = 1,691,036 eTable 4. Socio-Demographic Variations in Care Continuum in India Relative to Population With Hypertension, N = 1,691,036 eTable 5. Average Values of Systolic and Diastolic Blood Pressure for Each of the Three Measurements, N = 1,691,036 eTable 6. Socio-Demographic Disparities in Hypertension Care Continuum in India When Using Average of Last 2 Blood Pressure Measurements, N = 1,691,036 eFigure 1. Flowchart of Analytic Sample eFigure 2. Distribution of Diagnosed Hypertension by Treatment and Control Status eFigure 3. Distribution of Diagnosed Hypertension by Treatment and Control Status When Using Average of Last 2 Blood Pressure Measurements eFigure 4. State-Level Care Continuum eFigure 5. Heatmap for Care Continuum eFigure 6. Distribution of Between- and Within-State Variability in Hypertension Care Continua for Selected States eFigure 7. Disparities Within States at the District Level eMethods. National Family Health Survey-5, 2019-21: Survey Design and Data Collection [file jamanetwopen-e2339098-s001.pdf]

Supplementary Online Content

Varghese JS, Srinivasapura Venkateshmurthy N, Sudharsanan N, et al. Hypertension diagnosis, treatment, and control in India. *JAMA Netw Open.* 2023;6(10):e2339098. doi:10.1001/jamanetworkopen.2023.39098

eTable 1. Characteristics of Participants in Analytic Sample Versus Those Excluded, N = 1,895,297
eTable 2. Definitions of Disease and Care Continuum of Hypertension
eTable 3. Crude Estimates of Socio-Demographic Variations in Care Continuum in India Relative to Population With Hypertension, N = 1,691,036
eTable 4. Socio-Demographic Variations in Care Continuum in India Relative to Population With Hypertension, N = 1,691,036
eTable 5. Average Values of Systolic and Diastolic Blood Pressure for Each of the Three Measurements, N = 1,691,036
eTable 6. Socio-Demographic Disparities in Hypertension Care Continuum in India When Using Average of Last 2 Blood Pressure Measurements, N = 1,691,036
eFigure 1. Flowchart of Analytic Sample
eFigure 2. Distribution of Diagnosed Hypertension by Treatment and Control Status
eFigure 3. Distribution of Diagnosed Hypertension by Treatment and Control Status When Using Average of Last 2 Blood Pressure Measurements
eFigure 4. State-Level Care Continuum
eFigure 5. Heatmap for Care Continuum
eFigure 6. Distribution of Between- and Within-State Variability in Hypertension Care Continua for Selected States
eFigure 7. Disparities Within States at the District Level
eMethods. National Family Health Survey-5, 2019-21: Survey Design and Data Collection

This supplementary material has been provided by the authors to give readers additional information about their work.

© 2023 Varghese JS et al. *JAMA Network Open.*

eTable 1. Characteristics of Participants in Analytic Sample Versus Those Excluded, n = 1,895,297

	**Urban**		**Rural**	
	**Analytic Sample (n = 417,490)**	**Excluded Sample (n = 66,914)**	**Analytic Sample (n = 1,273,546)**	**Excluded Sample (n = 137,347)**
**Sex**				
Women	52.6 (52.4, 52.8)	36.4 (35.7, 37)	53.2 (53.1, 53.3)	34.6 (34.3, 35)
Men	47.4 (47.2, 47.6)	63.6 (63, 64.3)	46.8 (46.7, 46.9)	65.4 (65, 65.7)
**Age category**				
18-39	49.6 (49.2, 49.9)	55.8 (55.1, 56.5)	49.6 (49.4, 49.8)	59 (58.6, 59.3)
40-64	39.8 (39.5, 40)	35.9 (35.4, 36.5)	38.7 (38.6, 38.9)	32.2 (31.8, 32.5)
65 and above	10.7 (10.5, 10.9)	8.2 (7.8, 8.7)	11.6 (11.5, 11.7)	8.9 (8.7, 9.1)
**Schooling**				
None	16.2 (15.9, 16.6)	12.8 (12.2, 13.4)	33.3 (33.1, 33.6)	27.8 (27.4, 28.2)
Primary (up to 4th class)	12 (11.8, 12.2)	8.8 (8.4, 9.2)	15.6 (15.4, 15.7)	13.4 (13.2, 13.7)
Secondary (5th to 10th class)	46.9 (46.5, 47.2)	44.9 (44, 45.8)	40.7 (40.5, 40.9)	44.8 (44.4, 45.2)
Post-secondary (11th class and above)	24.9 (24.4, 25.4)	33.5 (32.2, 34.7)	10.4 (10.2, 10.6)	14 (13.7, 14.4)
**Caste of head of household**				
General or unspecified	35.1 (34.2, 36)	42.4 (40.7, 44.2)	23.4 (23, 23.9)	24.4 (23.7, 25.1)
Other Backward Castes	41.8 (41, 42.7)	38.1 (36.5, 39.6)	41.9 (41.5, 42.4)	42.8 (42, 43.5)
Scheduled Caste	19 (18.3, 19.7)	16.7 (15.6, 17.8)	22.6 (22.2, 23)	22.6 (22, 23.2)
Scheduled Tribe	4.1 (3.8, 4.4)	2.8 (2.5, 3.1)	12 (11.6, 12.3)	10.3 (9.8, 10.8)
**Religion**				
Hindu	78.6 (77.7, 79.5)	73.2 (71.4, 74.9)	84.3 (83.9, 84.8)	81.6 (80.8, 82.3)
Muslim	15.4 (14.5, 16.3)	19 (17.4, 20.7)	10.4 (10, 10.8)	12.5 (11.9, 13.2)
Other	6 (5.6, 6.3)	7.8 (6.8, 8.8)	5.3 (5.1, 5.5)	5.9 (5.5, 6.3)
**Household wealth quintile (by residence)**				
Lowest	19.4 (18.6, 20.1)	13.2 (12.2, 14.2)	17.8 (17.5, 18.1)	17.9 (17.3, 18.4)
Low	20.2 (19.7, 20.7)	15.7 (14.8, 16.6)	19.1 (18.9, 19.4)	18.5 (18.1, 19)
Medium	20.3 (19.9, 20.8)	18 (17.2, 18.9)	20.4 (20.1, 20.6)	18.6 (18.2, 19.1)

© 2023 Varghese JS et al. *JAMA Network Open.*

High	20.3 (19.8, 20.8)	22.2 (21.2, 23.2)	21.2 (20.9, 21.4)	20 (19.5, 20.5)
Highest	19.8 (19.1, 20.5)	30.8 (29, 32.6)	21.5 (21.2, 21.9)	25 (24.3, 25.7)
**Blood pressure measurement**				
Average Systolic BP	122.9 (122.8, 123.1)		122.1 (122.1, 122.2)	
Average Diastolic BP	80.9 (80.8, 81)		80.4 (80.3, 80.4)	
**Hypertension**				
Self-reported or high blood pressure	30 (29.6, 30.4)		26.6 (26.4, 26.8)	
Self-reported	13.2 (12.9, 13.5)		10 (9.8, 10.1)	

All values are percentages (95% confidence intervals).

© 2023 Varghese JS et al. *JAMA Network Open.*

eTable 2. Definitions of Disease and Care Continuum of Hypertension

Term	Study Population (Denominator)	Definition	Comment
Hypertension	Analytic sample	(a) Self-reported high blood pressure<br>OR (b) High blood pressure (≥140 mmHg systolic BP or ≥90 mmHg diastolic BP)<br><br>Defined as per ICMR guidelines with lowest of first 2 SBP measurements if difference <= 5mmHg or lowest of three measurements otherwise. Corresponding DBP measure was used, else the smallest available DBP measure.<br><br>Information on current medication status was asked only to those who reported having hypertension	Measurement of blood pressure at a single time point doesn't meet the confirmatory standards of diagnosis for hypertension (i.e. elevated BP at two or more sittings). Diagnosis requires a minimum of 2 sets of readings on 2 different occasions, which are at least 1-4 weeks apart.
Diagnosis	"Hypertension" as Yes	Told had high blood pressure on two or more occasions by a medical provider	Self-reported
Treatment	"Diagnosis" as Yes	Currently taking a prescribed medicine to lower blood pressure	Self-reported. We do not have information on dosage and type of medication used
Control	"Treatment" as Yes	Blood pressure in non-hypertensive range (<140/90 mmHg if age < 80 years and <150/90 mmHg if age ≥ 80 years)	

© 2023 Varghese JS et al. *JAMA Network Open.*

eTable 3. Crude Estimates of Socio-Demographic Variations in Care Continuum in India Relative to Population With Hypertension, N = 1,691,036

	**Total**				**Urban**				**Rural**			
	**Hypertension (%)**	**Diagnosed [a] (%)**	**Treated [a] (%)**	**Controlled [a] (%)**	**Hypertension (%)**	**Diagnosed [a] (%)**	**Treated [a] (%)**	**Controlled [a] (%)**	**Hypertension (%)**	**Diagnosed [a] (%)**	**Treated [a] (%)**	**Controlled [a] (%)**
**Total**	27.7 (27.5, 27.9)	39.6 (39.2, 40.1)	58.4 (57.7, 59)	45.4 (44.9, 45.9)	30 (29.6, 30.4)	43.9 (43.1, 44.7)	67.3 (66, 68.5)	45.9 (45, 46.8)	26.6 (26.4, 26.8)	37.4 (36.9, 37.9)	52.8 (52, 53.6)	45 (44.5, 45.6)
**Sex**												
Women	27.1 (26.9, 27.3)	46.6 (46.2, 47.1)	57.9 (57.2, 58.6)	46.4 (45.7, 47)	29.1 (28.7, 29.4)	51.5 (50.6, 52.4)	66.9 (65.7, 68.2)	47 (46, 48.1)	26.2 (26, 26.4)	44.1 (43.6, 44.7)	52.5 (51.6, 53.3)	45.8 (45.1, 46.5)
Men	28.4 (28.1, 28.6)	32.1 (31.7, 32.5)	59.1 (58.3, 59.9)	44 (43.2, 44.7)	31 (30.6, 31.5)	36 (35.2, 36.8)	67.8 (66.3, 69.2)	44.2 (42.9, 45.5)	27.1 (26.9, 27.3)	30 (29.5, 30.5)	53.4 (52.4, 54.3)	43.8 (42.9, 44.7)
**Age category**												
18-39	14.3 (14.1, 14.4)	30.6 (29.9, 31.2)	22.9 (22.1, 23.7)	61.3 (59.7, 62.8)	14.9 (14.5, 15.2)	28.4 (27.1, 29.7)	26.4 (24.7, 28.1)	56.8 (53.9, 59.7)	14 (13.8, 14.2)	31.7 (30.9, 32.4)	21.3 (20.4, 22.2)	63.8 (62, 65.6)
40-64	37.3 (37, 37.5)	39.6 (39.2, 40)	61.9 (61.2, 62.6)	43.7 (43, 44.3)	40.9 (40.3, 41.4)	44.7 (43.9, 45.5)	70.3 (69.1, 71.4)	44.6 (43.5, 45.6)	35.6 (35.3, 35.8)	36.8 (36.3, 37.3)	56.2 (55.4, 57)	42.9 (42.2, 43.7)
65 and above	53.3 (53, 53.7)	50.4 (49.9, 51)	76.9 (76.3, 77.6)	44.7 (43.9, 45.5)	59.7 (58.9, 60.5)	59.9 (58.8, 61)	84 (82.9, 85)	46.1 (44.7, 47.5)	50.6 (50.2, 51)	45.6 (45, 46.2)	72.2 (71.4, 73)	43.6 (42.7, 44.5)
**Schooling**												
None	34.3 (34, 34.6)	39.2 (38.7, 39.7)	58.8 (58, 59.6)	42.1 (41.4, 42.9)	39.8 (39.1, 40.5)	45.6 (44.5, 46.8)	68.4 (66.7, 70.1)	42 (40.4, 43.7)	33 (32.8, 33.3)	37.5 (36.9, 38)	55.6 (54.7, 56.5)	42.2 (41.3, 43)

© 2023 Varghese JS et al. *JAMA Network Open.*

Primary (up to 4th class)	31.9 (31.5, 32.2)	41.1 (40.5, 41.7)	63.9 (63, 64.9)	45.6 (44.6, 46.6)	37.4 (36.6, 38.2)	45.8 (44.6, 47.1)	72.8 (71.1, 74.5)	45.3 (43.6, 47)	29.8 (29.5, 30.2)	38.9 (38.2, 39.6)	59.2 (58, 60.3)	45.8 (44.5, 47)
Secondary (5th to 10th class)	24.5 (24.2, 24.7)	39.2 (38.7, 39.7)	56.6 (55.8, 57.5)	46.9 (46, 47.7)	28.5 (28.1, 28.9)	42.8 (41.9, 43.7)	66.8 (65.4, 68.2)	46.4 (45.1, 47.7)	22.3 (22, 22.5)	36.7 (36.1, 37.3)	48.4 (47.4, 49.4)	47.4 (46.4, 48.5)
Post-secondary (11th class and above)	20.6 (20.3, 21)	40.3 (39.4, 41.2)	54.3 (52.8, 55.8)	50.8 (49.2, 52.5)	22.8 (22.2, 23.3)	43 (41.6, 44.3)	62.4 (60.3, 64.4)	50.3 (48.2, 52.3)	18.2 (17.7, 18.7)	36.5 (35.4, 37.5)	40.9 (39.1, 42.7)	52.3 (49.8, 54.7)
**Household wealth quintile**												
Lowest	24.9 (24.6, 25.2)	32.3 (31.5, 33)	47.1 (45.7, 48.5)	43.7 (42.2, 45.1)	26.8 (26.1, 27.4)	37.2 (35.7, 38.7)	55.8 (53.1, 58.5)	41.9 (39.7, 44.1)	23.9 (23.6, 24.2)	29.4 (28.6, 30.3)	40.7 (39.2, 42.2)	45.5 (43.6, 47.3)
Low	25.7 (25.4, 26)	37.1 (36.4, 37.7)	53 (51.9, 54)	44.2 (43.1, 45.4)	28.8 (28.3, 29.4)	41.4 (40.2, 42.6)	64.6 (62.7, 66.5)	44.2 (42.4, 45.9)	24.2 (23.9, 24.5)	34.5 (33.8, 35.2)	44.6 (43.4, 45.9)	44.3 (42.8, 45.7)
Medium	27.1 (26.8, 27.4)	39.1 (38.4, 39.7)	57.9 (56.8, 58.9)	43.8 (42.7, 44.9)	30 (29.4, 30.5)	43.8 (42.6, 44.9)	69.6 (67.9, 71.4)	44.5 (42.7, 46.4)	25.8 (25.5, 26.1)	36.5 (35.8, 37.2)	50.2 (49, 51.4)	43.1 (41.8, 44.4)
High	28.6 (28.3, 28.9)	41.3 (40.7, 41.9)	62.5 (61.6, 63.5)	46 (45.1, 47)	31 (30.5, 31.6)	45.8 (44.6, 46.9)	70.3 (68.6, 72)	47.1 (45.3, 48.8)	27.5 (27.1, 27.8)	39 (38.3, 39.7)	57.9 (56.7, 59.1)	45.3 (44.2, 46.4)
Highest	31.7 (31.3, 32)	45.7 (45, 46.3)	64.1 (63.2, 65)	47.2 (46.3, 48.1)	33.3 (32.6, 33.9)	49.7 (48.4, 51)	71.4 (69.7, 73.1)	48.9 (47.2, 50.5)	31 (30.6, 31.3)	43.8 (43.1, 44.5)	60.2 (59.2, 61.3)	46.1 (45.2, 47.1)

© 2023 Varghese JS et al. *JAMA Network Open.*

eTable 4. Socio-Demographic Variations in Care Continuum in India Relative to Population With Hypertension, N = 1,691,036

	**Total**				**Urban**				**Rural**			
	**Hypertension (%)**	**Diagnosed [a] (%)**	**Treated [a] (%)**	**Controlled [a] (%)**	**Hypertension (%)**	**Diagnosed [a] (%)**	**Treated [a] (%)**	**Controlled [a] (%)**	**Hypertension (%)**	**Diagnosed [a] (%)**	**Treated [a] (%)**	**Controlled [a] (%)**
**Total**	28.1 (27.9, 28.3)	36.9 (36.4, 37.3)	17.7 (17.5, 18)	8.5 (8.3, 8.6)	32.6 (32.2, 33)	39.9 (39.1, 40.8)	24 (23.4, 24.5)	11.2 (10.8, 11.5)	25.9 (25.7, 26.1)	35.4 (34.8, 35.9)	14.7 (14.4, 14.9)	7.1 (7, 7.3)
**Sex**												
Women	25.7 (25.5, 25.9)	44.6 (44, 45.1)	20.2 (19.8, 20.5)	10.1 (9.8, 10.3)	30.1 (29.7, 30.5)	47.9 (46.8, 48.9)	27.5 (26.8, 28.2)	13.3 (12.8, 13.8)	23.7 (23.5, 23.9)	43.1 (42.4, 43.8)	16.9 (16.5, 17.2)	8.6 (8.3, 8.9)
Men	30.6 (30.3, 30.8)	28.4 (27.9, 28.8)	15.1 (14.8, 15.3)	6.7 (6.5, 6.9)	35.1 (34.6, 35.6)	32.2 (31.4, 33)	20.5 (19.9, 21)	9.1 (8.7, 9.5)	28.2 (27.9, 28.5)	26.3 (25.8, 26.8)	12.1 (11.9, 12.4)	5.4 (5.2, 5.6)
**Age category**												
18-39	14.9 (14.8, 15.1)	31.5 (30.8, 32.2)	7.4 (7.1, 7.7)	4.5 (4.3, 4.7)	15.6 (15.2, 15.9)	28.6 (27.2, 29.9)	7.6 (7.1, 8.2)	4.3 (3.9, 4.8)	14.7 (14.5, 14.9)	32.6 (31.8, 33.4)	7.3 (7, 7.6)	4.6 (4.3, 4.8)
40-64	37.2 (36.9, 37.5)	39.5 (39.1, 40)	24.5 (24.1, 24.8)	10.7 (10.5, 10.9)	40.2 (39.6, 40.7)	44.5 (43.7, 45.3)	31.2 (30.5, 31.8)	13.9 (13.5, 14.4)	35.4 (35.1, 35.7)	36.6 (36.1, 37.1)	20.5 (20.1, 20.8)	8.8 (8.6, 9)
65 and above	54.3 (53.8, 54.8)	51.3 (50.7, 51.9)	39.8 (39.2, 40.4)	17.9 (17.5, 18.4)	60.1 (59.1, 61.1)	59.8 (58.6, 61)	50.3 (49.1, 51.5)	23 (22.1, 24)	50.4 (49.9, 50.9)	45.7 (45.1, 46.4)	32.9 (32.3, 33.5)	14.6 (14.1, 15)
**Schooling**												
None	27.6 (27.4, 27.9)	36.3 (35.6, 37)	15.6 (15.2, 15.9)	7.2 (7, 7.5)	32.8 (32.1, 33.5)	39 (37.4, 40.6)	20.8 (19.8, 21.8)	9.2 (8.5, 9.9)	26.5 (26.2, 26.8)	35.6 (34.8, 36.4)	14.3 (13.9, 14.6)	6.8 (6.5, 7)

© 2023 Varghese JS et al. *JAMA Network Open.*

Primary (up to 4th class)	28.8 (28.5, 29.1)	36 (35.3, 36.7)	18.2 (17.8, 18.7)	8.7 (8.4, 9)	34.8 (34, 35.5)	38.3 (36.9, 39.6)	23.9 (22.9, 25)	10.9 (10.2, 11.5)	26.7 (26.4, 27)	35.1 (34.3, 36)	16 (15.4, 16.5)	7.8 (7.5, 8.2)
Secondary (5th to 10th class)	28.1 (27.9, 28.3)	37 (36.5, 37.5)	18.6 (18.3, 18.9)	8.9 (8.6, 9.1)	32.9 (32.5, 33.4)	40 (39.1, 40.9)	24.7 (24.1, 25.4)	11.5 (11.1, 12)	25.3 (25.1, 25.6)	35.2 (34.5, 35.8)	14.7 (14.3, 15)	7.2 (6.9, 7.4)
Post-secondary (11th class and above)	28.2 (27.8, 28.7)	39.4 (38.6, 40.3)	20.8 (20.2, 21.4)	10.5 (10, 10.9)	31 (30.3, 31.7)	42.1 (40.8, 43.3)	25.7 (24.7, 26.6)	12.7 (12, 13.4)	24.4 (23.8, 25)	35.8 (34.7, 36.9)	14.1 (13.5, 14.8)	7.4 (6.9, 7.9)
**Household wealth quintile**												
Lowest	25.4 (25.1, 25.8)	32 (31.1, 32.9)	12.2 (11.8, 12.6)	5.8 (5.5, 6.1)	29 (28.3, 29.8)	34.9 (33.1, 36.6)	16.3 (15.4, 17.1)	7.1 (6.6, 7.7)	23.6 (23.2, 23.9)	30.4 (29.4, 31.4)	9.9 (9.4, 10.3)	5.1 (4.7, 5.5)
Low	26.7 (26.4, 27)	35.9 (35.1, 36.6)	15.8 (15.4, 16.2)	7.4 (7.1, 7.7)	32 (31.3, 32.6)	38.2 (36.9, 39.5)	22.2 (21.3, 23.1)	9.9 (9.3, 10.5)	23.9 (23.6, 24.2)	34.5 (33.6, 35.5)	12.2 (11.7, 12.6)	6 (5.7, 6.4)
Medium	27.9 (27.6, 28.2)	36.8 (36.1, 37.5)	17.6 (17.2, 18)	8.1 (7.8, 8.5)	33.3 (32.6, 33.9)	39.7 (38.5, 40.9)	25.2 (24.2, 26.1)	11.5 (10.8, 12.2)	25.1 (24.8, 25.5)	35.4 (34.4, 36.3)	13.8 (13.3, 14.2)	6.4 (6.1, 6.8)
High	29 (28.7, 29.3)	37.3 (36.5, 38)	19.4 (18.9, 19.8)	9.3 (9, 9.6)	33.9 (33.2, 34.6)	41.7 (40.4, 43)	26.7 (25.7, 27.7)	12.7 (11.9, 13.4)	26.6 (26.3, 27)	35.2 (34.3, 36.1)	16 (15.6, 16.5)	7.7 (7.4, 8.1)
Highest	31.1 (30.7, 31.4)	40.7 (39.9, 41.4)	21.7 (21.2, 22.1)	10.5 (10.2, 10.9)	34.8 (34, 35.5)	44.3 (42.8, 45.8)	28.3 (27.2, 29.4)	14 (13.2, 14.8)	29.4 (29.1, 29.8)	39.2 (38.3, 40.1)	19 (18.4, 19.5)	9.1 (8.7, 9.5)

Estimates (95% confidence intervals) are standardized to age distribution in overall sample. We performed age-standardization to the distribution of the within-sample total population separately for total population (**Supplementary Table 3**), hypertension (**Table 2**), diagnosed population (**Table 2**) and treated (**Table 2**)population

a Among those with self-reported hypertension or high blood pressure (≥140/90 mmHg).

© 2023 Varghese JS et al. *JAMA Network Open.*

eTable 5. Average Values of Systolic and Diastolic Blood Pressure for Each of the Three Measurements, N = 1,691,036

	**SBP 1**	**SBP 2**	**SBP 3**	**DBP 1**	**DBP 2**	**DBP 3**
Total	127.1 (127, 127.2)	124.5 (124.5, 124.6)	122.9 (122.8, 122.9)	82.8 (82.8, 82.9)	81.2 (81.1, 81.2)	80.1 (80, 80.1)
Women	124.3 (124.3, 124.4)	121.7 (121.6, 121.8)	120 (119.9, 120.1)	81.7 (81.6, 81.7)	80 (79.9, 80)	78.9 (78.8, 78.9)
Men	130 (129.9, 130.1)	127.5 (127.4, 127.6)	125.9 (125.8, 126)	84.1 (84, 84.1)	82.4 (82.4, 82.5)	81.4 (81.3, 81.4)
Rural	126.4 (126.3, 126.4)	123.7 (123.7, 123.8)	122.1 (122, 122.2)	82.5 (82.4, 82.5)	80.8 (80.8, 80.9)	79.7 (79.7, 79.8)
Urban	128.6 (128.5, 128.8)	126.1 (126, 126.3)	124.4 (124.2, 124.6)	83.6 (83.5, 83.7)	81.9 (81.8, 82)	80.8 (80.7, 80.9)
ICC[a]		0.89				

All estimates of average blood pressure (in mm Hg) are age-standardized.

a Intra-class correlation coefficient from linear mixed model

© 2023 Varghese JS et al. *JAMA Network Open.*

eTable 6. Socio-Demographic Disparities in Hypertension Care Continuum in India When Using Average of Last 2 Blood Pressure Measurements, N = 1,691,036

	**Total**				**Urban**				**Rural**			
	**Hypertension (%)**	**Diagnosed [a] (%)**	**Treated [b] (%)**	**Controlled [c] (%)**	**Hypertension (%)**	**Diagnosed [a] (%)**	**Treated [b] (%)**	**Controlled [c] (%)**	**Hypertension (%)**	**Diagnosed [a] (%)**	**Treated [b] (%)**	**Controlled [c] (%)**
**Total**	27.1 (27, 27.3)	38.9 (38.4, 39.4)	44.5 (43.9, 45.1)	53.1 (52.2, 53.9)	31.6 (31.2, 32)	41.7 (40.8, 42.6)	56.1 (54.7, 57.5)	51.3 (49.8, 52.8)	24.9 (24.7, 25.1)	37.5 (36.9, 38.1)	38.6 (37.8, 39.4)	54.3 (53.2, 55.3)
**Sex**												
Women	24.9 (24.7, 25.1)	46.9 (46.3, 47.6)	42 (41.3, 42.7)	56.3 (55.2, 57.3)	29.1 (28.7, 29.6)	50 (48.9, 51.1)	53.8 (52.3, 55.3)	53.9 (52.2, 55.7)	22.9 (22.7, 23.1)	45.6 (44.9, 46.3)	36.7 (35.8, 37.6)	57.8 (56.5, 59.1)
Men	29.6 (29.3, 29.8)	29.8 (29.3, 30.3)	49.2 (48.4, 50)	47.7 (46.3, 49.1)	34.1 (33.6, 34.6)	33.5 (32.6, 34.4)	59.7 (58.1, 61.4)	47.5 (45.2, 49.8)	27.2 (27, 27.5)	27.8 (27.3, 28.4)	42.8 (41.7, 43.8)	47.9 (46.2, 49.5)
**Age category**												
18-39	13.9 (13.7, 14.1)	34.4 (33.6, 35.1)	23.7 (22.8, 24.5)	62.2 (60.6, 63.8)	14.5 (14.1, 14.9)	31 (29.6, 32.5)	27.1 (25.2, 29)	59.2 (56.2, 62.2)	13.6 (13.4, 13.8)	35.7 (34.8, 36.5)	22.5 (21.5, 23.5)	63.9 (62.1, 65.7)
40-64	36.2 (35.9, 36.4)	40.9 (40.5, 41.3)	61.5 (60.9, 62.2)	44.1 (43.5, 44.8)	39.1 (38.5, 39.6)	46 (45.1, 46.8)	69.8 (68.6, 71)	45.1 (44, 46.2)	34.4 (34.1, 34.7)	37.9 (37.4, 38.4)	55.7 (54.9, 56.6)	43.3 (42.5, 44.1)
65 and above	54.1 (53.5, 54.6)	51.7 (51.1, 52.3)	77 (76.3, 77.7)	44 (43.2, 44.8)	59.8 (58.8, 60.8)	60.6 (59.4, 61.8)	83.8 (82.7, 84.9)	45.5 (44.1, 47)	50.3 (49.8, 50.8)	46 (45.3, 46.6)	71.6 (70.7, 72.4)	42.8 (41.9, 43.7)
**Schooling**												
None	26.7 (26.5, 27)	38.3 (37.6, 39.1)	41.4 (40.5, 42.4)	48.2 (47.1, 49.4)	31.8 (31.1, 32.5)	40.6 (38.9, 42.4)	52 (49.4, 54.6)	45.3 (42.8, 47.8)	25.6 (25.4, 25.9)	37.7 (36.9, 38.6)	38.6 (37.5, 39.7)	49.3 (48, 50.7)

© 2023 Varghese JS et al. *JAMA Network Open.*

Primary (up to 4th class)	27.8 (27.5, 28.1)	38 (37.2, 38.8)	46.7 (45.5, 47.9)	51 (49.2, 52.7)	33.8 (33, 34.5)	39.9 (38.5, 41.4)	58.6 (56.2, 61.1)	47.3 (44.5, 50)	25.7 (25.4, 26.1)	37.2 (36.3, 38.1)	42 (40.5, 43.4)	52.9 (50.7, 55.1)
Secondary (5th to 10th class)	27.2 (26.9, 27.4)	39 (38.5, 39.6)	45.6 (44.9, 46.4)	53.4 (52, 54.7)	31.9 (31.5, 32.4)	41.8 (40.8, 42.8)	57.7 (56.1, 59.2)	49.9 (47.8, 52)	24.4 (24.2, 24.7)	37.3 (36.6, 37.9)	37.7 (36.8, 38.7)	56 (54.3, 57.8)
Post-secondary (11th class and above)	27.2 (26.8, 27.7)	41.6 (40.7, 42.5)	46.7 (45.5, 47.8)	61.2 (58.8, 63.7)	30.1 (29.4, 30.8)	44 (42.6, 45.3)	55.3 (53.4, 57.2)	60.1 (56.8, 63.4)	23.4 (22.8, 24)	38.4 (37.2, 39.5)	34.3 (32.7, 36)	63 (59.2, 66.8)
**Household wealth quintile**												
Lowest	24.5 (24.2, 24.8)	34 (33.1, 34.9)	36.9 (35.7, 38.1)	55.5 (53.5, 57.5)	28 (27.3, 28.7)	36.9 (35.1, 38.7)	44.8 (42.1, 47.5)	51.3 (48.1, 54.5)	22.7 (22.4, 23)	32.4 (31.3, 33.5)	31.9 (30.4, 33.4)	58.8 (56.2, 61.5)
Low	25.7 (25.4, 26)	38 (37.3, 38.8)	41.6 (40.6, 42.7)	53 (51.3, 54.8)	30.9 (30.2, 31.6)	40 (38.7, 41.4)	55.1 (53, 57.3)	48.3 (45.5, 51.1)	23 (22.7, 23.3)	36.9 (36, 37.9)	33.7 (32.4, 35)	57.2 (55, 59.4)
Medium	27 (26.7, 27.3)	38.9 (38.1, 39.6)	44.3 (43.3, 45.4)	53.8 (51.9, 55.6)	32.3 (31.6, 32.9)	41.6 (40.3, 42.9)	59.5 (57.4, 61.6)	51.5 (48.4, 54.5)	24.3 (24, 24.6)	37.5 (36.5, 38.4)	36.4 (35.1, 37.7)	55.6 (53.3, 57.9)
High	28.1 (27.8, 28.4)	39.1 (38.4, 39.9)	47.1 (46, 48.2)	52.8 (51.1, 54.6)	33 (32.3, 33.7)	43 (41.6, 44.4)	59.2 (57, 61.3)	51.6 (48.6, 54.6)	25.7 (25.3, 26)	37.3 (36.4, 38.3)	41.2 (39.9, 42.6)	53.6 (51.5, 55.7)
Highest	30 (29.7, 30.4)	42.7 (41.9, 43.5)	47.9 (46.8, 49)	52 (50.3, 53.6)	33.8 (33, 34.5)	46 (44.4, 47.6)	58.5 (56.1, 60.8)	52.9 (49.7, 56.2)	28.4 (28, 28.7)	41.3 (40.4, 42.2)	43.6 (42.2, 45)	51.4 (49.5, 53.4)

Estimates (95% confidence intervals) are standardized to age distribution in overall sample.
a Among those with self-reported high blood pressure or high measured blood pressure (≥140/90 mmHg).
b Among those with self-reported hypertension (‘Diagnosed’)
c Among those taking medication for hypertension (‘Treated’)

© 2023 Varghese JS et al. *JAMA Network Open.*

eFigure 1. Flowchart of Analytic Sample

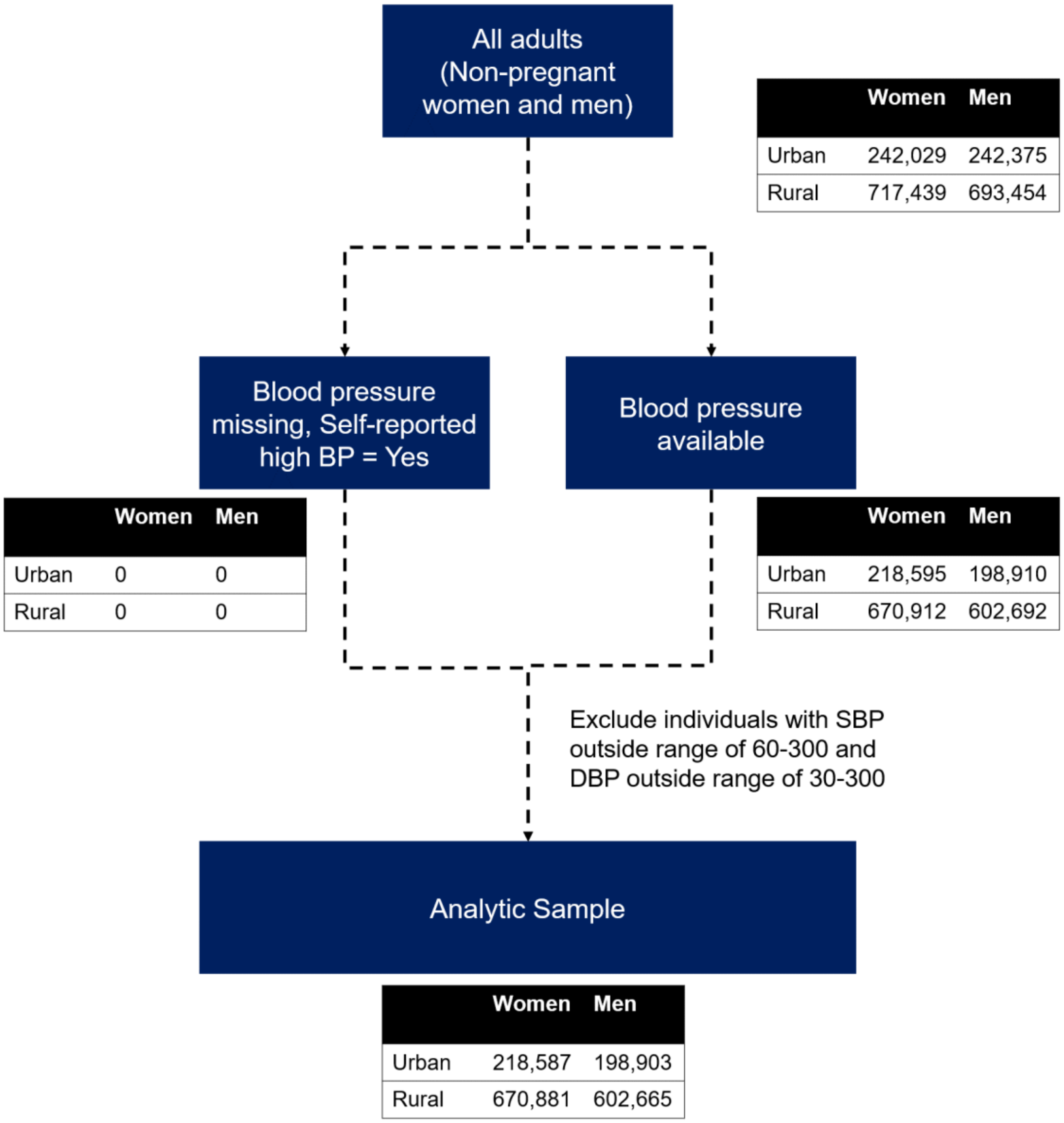

© 2023 Varghese JS et al. *JAMA Network Open.*

eFigure 2. Distribution of Diagnosed Hypertension by Treatment and Control Status

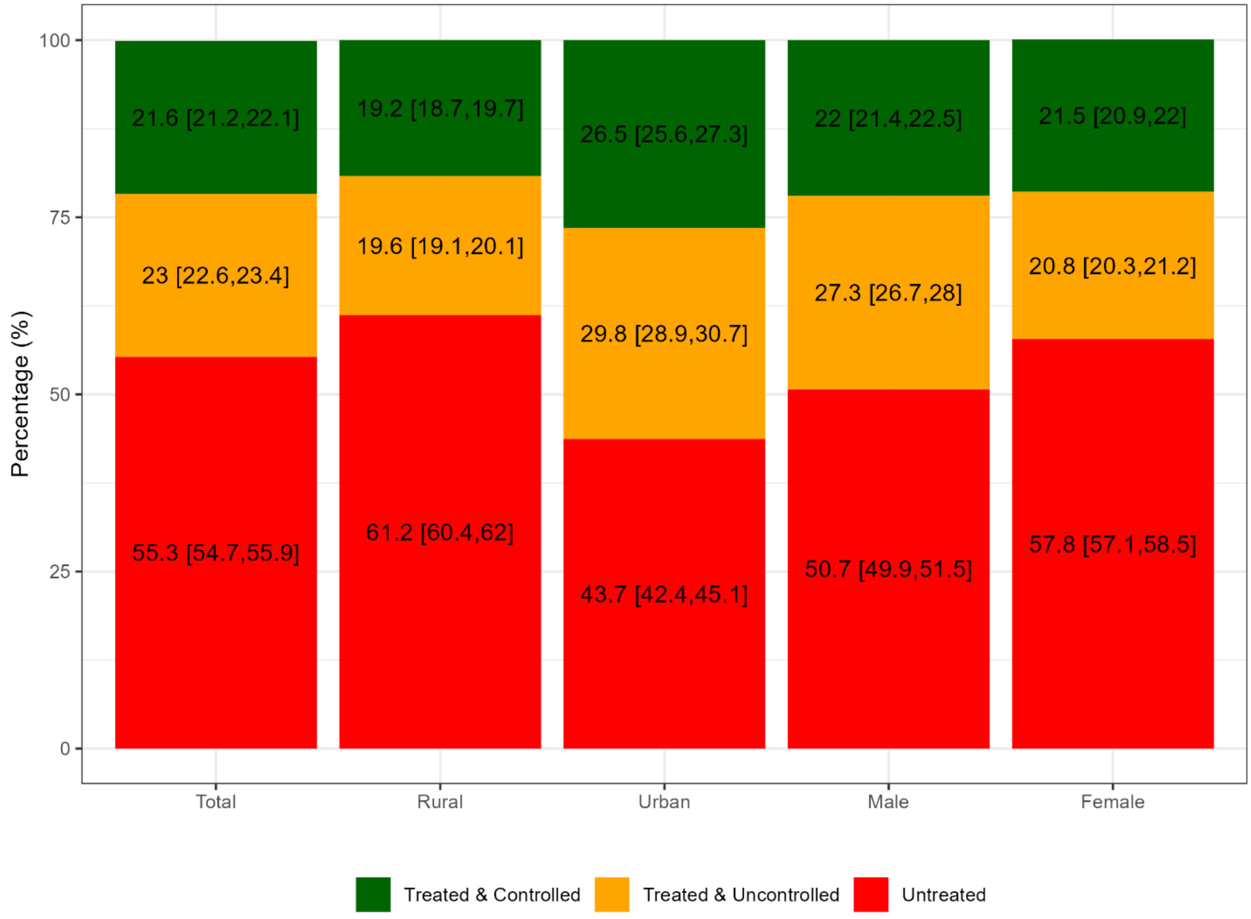

All estimates (95% confidence intervals) are age standardized proportions among those who self-reported diagnosis of hypertension (n = 179,322).

© 2023 Varghese JS et al. *JAMA Network Open.*

eFigure 3. Distribution of Diagnosed Hypertension by Treatment and Control Status When Using Average of Last 2 Blood Pressure Measurements

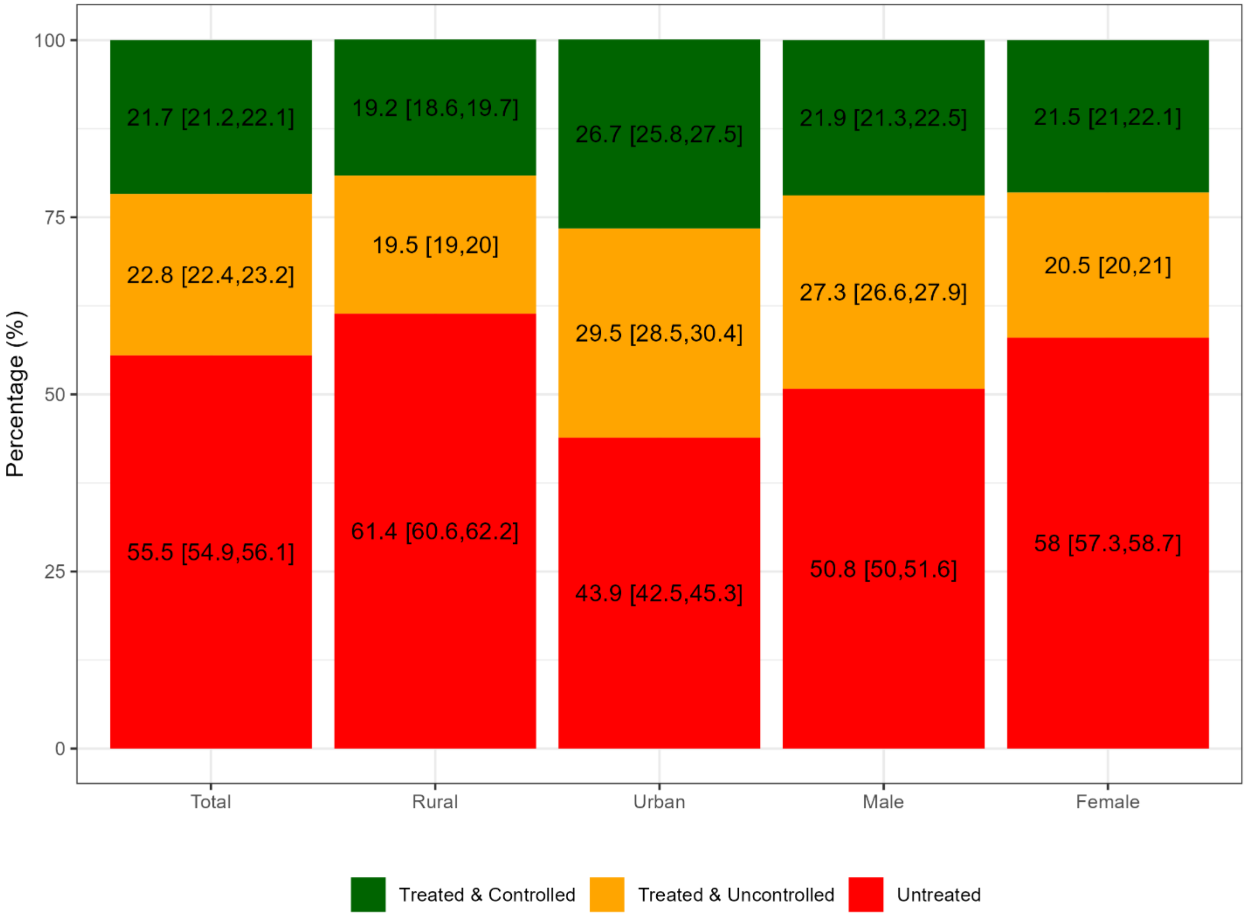

All estimates (95% confidence intervals) are age standardized proportions among those who self-reported diagnosis of hypertension (n = 179,322).

© 2023 Varghese JS et al. *JAMA Network Open.*

eFigure 4. State-Level Care Continuum

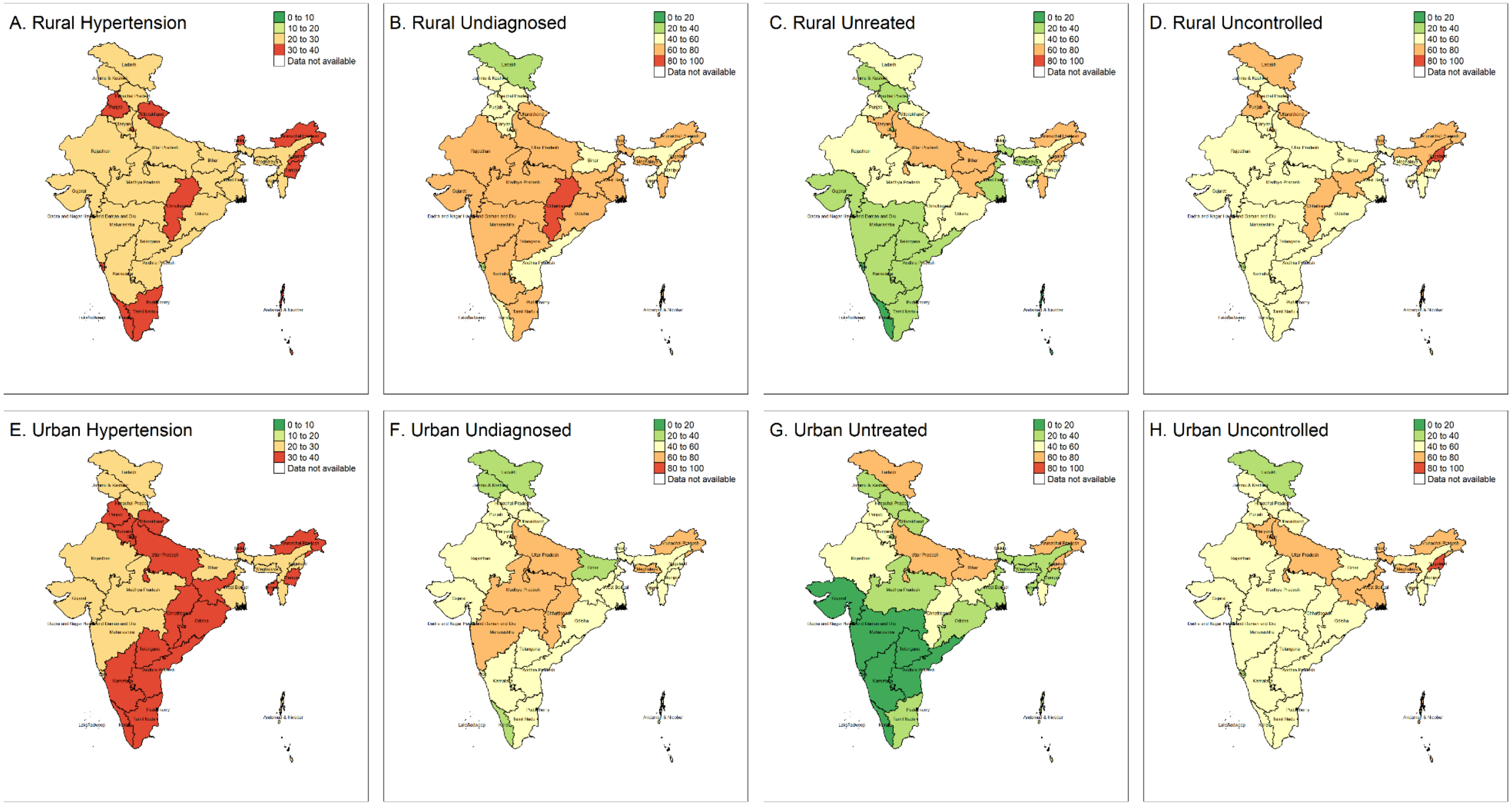

All values are survey weighted percentages (not age standardized). Refer to dashboard for detailed presentation; Undiagnosed are among those with hypertension (n = 470,020). Untreated and uncontrolled are among those diagnosed with hypertension and among those treated respectively.

© 2023 Varghese JS et al. *JAMA Network Open.*

eFigure 5. Heatmap for Care Continuum

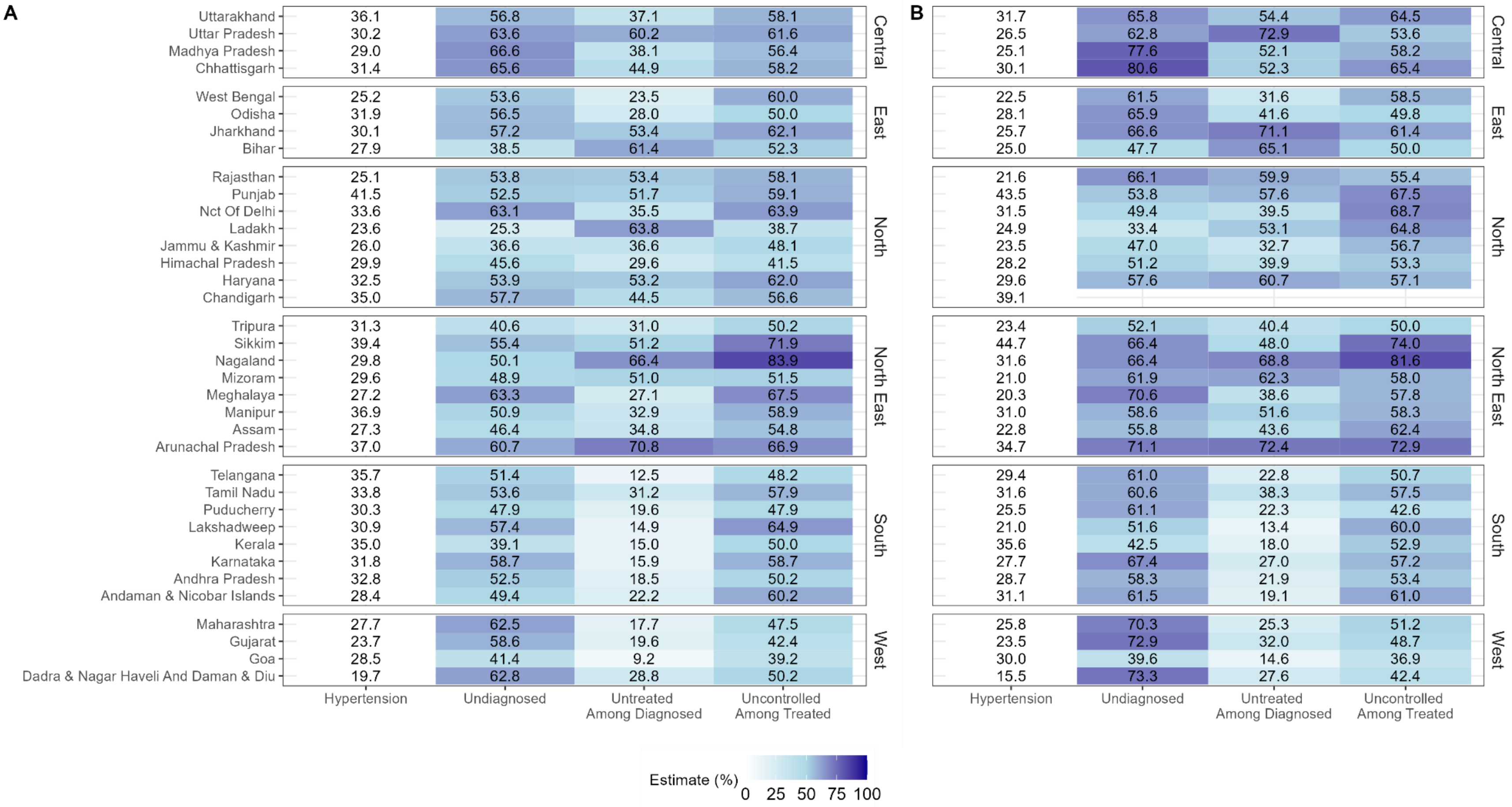

A. Urban, B. Rural. All values are survey weighted percentages (not age standardized) and 95% confidence intervals accounting for survey design. Undiagnosed are among those with hypertension (n = 470,020). Untreated and uncontrolled are among those diagnosed with hypertension and among those treated respectively.

© 2023 Varghese JS et al. *JAMA Network Open.*

eFigure 6. Distribution of Between- and Within-State Variability in Hypertension Care Continua for Selected States

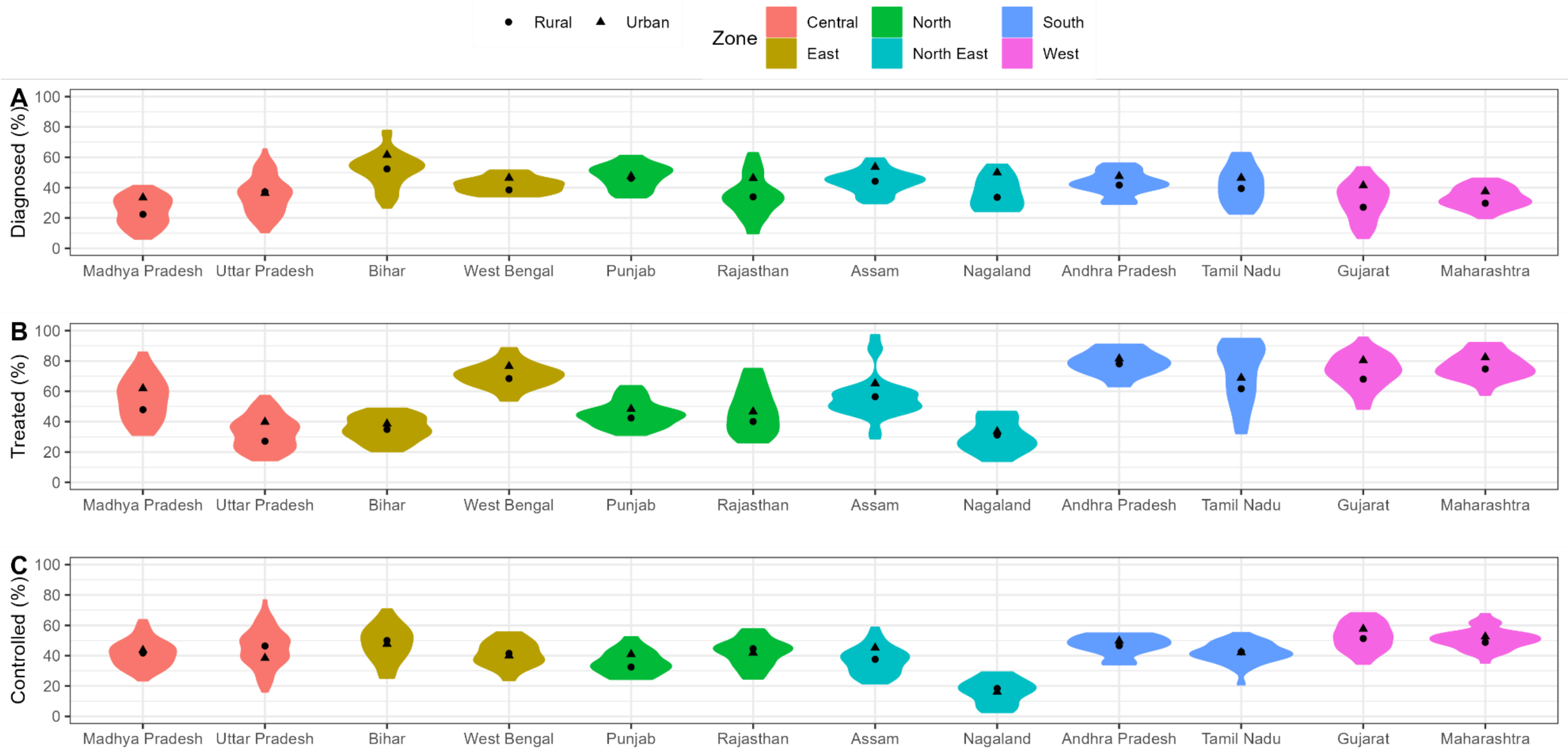

All values are survey weighted, crude percentages (not age standardized) of care continua represented as a violin plot for 12 states from 6 administrative zones, along with state-level rural and urban estimates. Width of each violin represents relative density of points. An elongated violin suggests greater within-state (between-district) variability in care continua. A: Proportion diagnosed among those with hypertension; B: Proportion treated among diagnosed hypertension; C: Proportion controlled among treated hypertension.

© 2023 Varghese JS et al. *JAMA Network Open.*

eFigure 7. Disparities Within States at the District Level

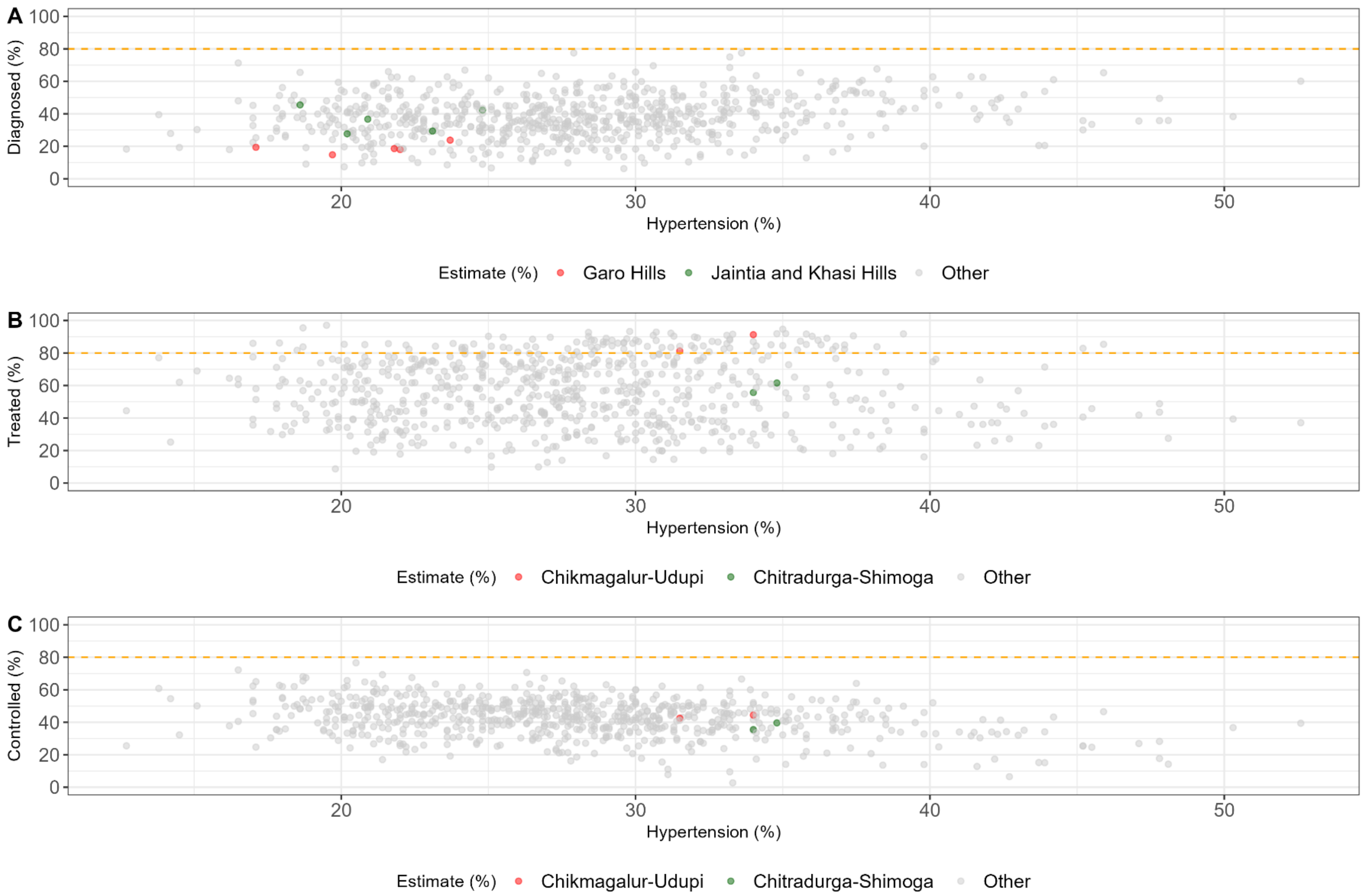

© 2023 Varghese JS et al. *JAMA Network Open.*

All values are survey weighted percentages (not age standardized)

© 2023 Varghese JS et al. *JAMA Network Open.*

eMethods. National Family Health Survey-5, 2019-21: Survey Design and Data Collection

The National Family Health Survey-5, 2019-21 is a multistage sampling survey (see *Survey Design*) that collected information on population demographics, health and nutritional status for 36 states and 707 districts. The fieldwork for the survey was conducted in two phases. Phase 1 was conducted from 17 June 2019 to 30 January 2020, and covered 342 districts from 17 states and 5 Union Territories completely, and 88 districts from the other 14 states. Phase 2 was conducted from 21 November 2020 to 30 April 2021, when the remaining 277 (707 minus (342 + 88)) districts from the other 14 states were completed. In total, the survey enumerators gathered information on 636,699 households comprising 724,115 women 15-49 years and 101,839 men 15-54 years. NFHS-5 used a multi-stage stratified sample where primary sampling units (PSUs) from urban (census enumeration blocks) and rural (villages) strata of each district were sampled at the first stage and households within PSUs were randomly sampled from a list of eligible households at the second stage.

Sample size

NFHS-5 was designed to provide district-level estimates for several demographic, health and nutrition indicators for reproductive age population and children under 5 years. The eligible sample (women 15-49 years, men 15-54 years) was consistent with the previous round, i.e. NFHS-4, to allow for studying trends in indicators. Sample sizes for NFHS-5 were worked out based on estimating the proportion of women of reproductive age (15-49 years) who had 3 or more antenatal care visits as the key indicator, with NFHS-4 estimates used as the reference for different states. Unlike the previous round (NFHS-4), NFHS-5 enumerators collected glucose and blood pressure were measured for all women and men age 15 and above in the households of eligible sample (see *Data Availability* below).

eReferences.

1. International Institute for Population Sciences, ICF. National Family Health Survey (NFHS-5), 2019-21: India. Mumbai, India: IIPS; 2022. https://dhsprogram.com/pubs/pdf/FR375/FR375.pdf
2. International Institute for Population Sciences. Clinical Anthropometric Biochemical (CAB) Manual. Mumbai: International Institute for Population Sciences; 2019 October 2019. rchiips.org/NFHS/NFHS5/manuals/NFHS-5 CAB Manual_Eng.pdf

© 2023 Varghese JS et al. *JAMA Network Open.*

Survey design

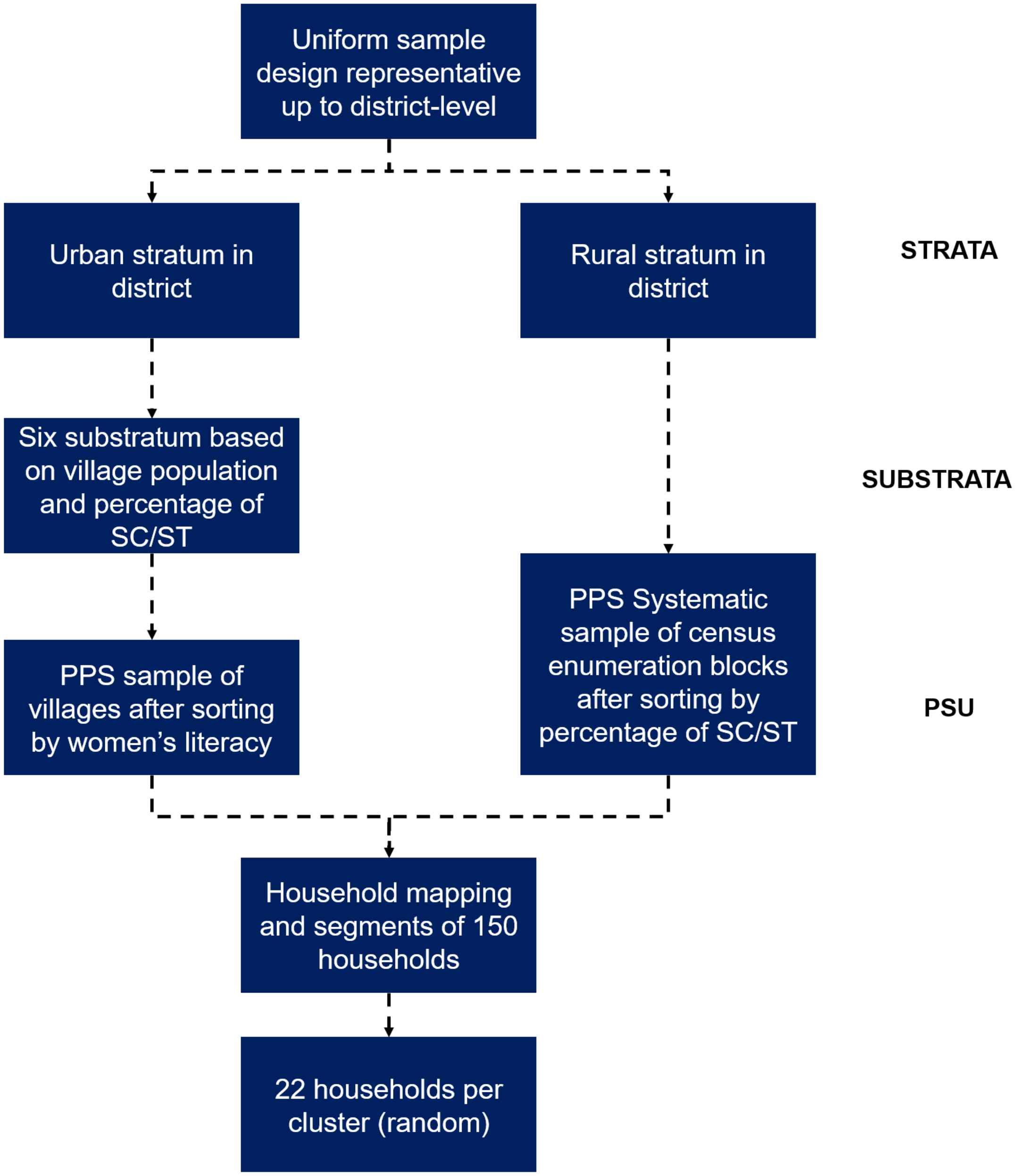

SC/ST: Scheduled castes or scheduled tribes are historically disadvantaged minority communities

PPS: Probability proportional to size

© 2023 Varghese JS et al. *JAMA Network Open.*

PSU: 30,198 small geographically defined areas were selected throughout the country as primary sampling units

Data Availability

	**Eligible Sample**		**Other adults in households where eligible participants resided**	
	**Women 15-49 years**	**Men 15-54 years**	**Women 15-98 years**	**Men 15-98 years**
Sample size	**724,115**	**101,839**	**340,355**	**911,737**
Non-pregnant and over 18 years	622,823	91,628	336,684	844,213
Available in Person Recode	622,784	91,616	336,684	844,213
Demographics	Detailed	Detailed	Basic (Age, sex, education etc.)	Basic (Age, sex, education etc.)
Medical History	Self-reported conditions* and medication status	Self-reported conditions* and medication status	Self-reported high blood glucose and high blood pressure, and medication status	Self-reported high blood glucose and high blood pressure, and medication status
Height, weight	Yes	Yes	No	No
Waist and Hip circumference	Yes	Yes	No	No
Blood pressure	Yes	Yes	Yes	Yes
Random Blood glucose with self-reported fasting duration	Yes	Yes	Yes	Yes
* diabetes, hypertension, chronic respiratory disease, thyroid disorder, heart disease, cancer, chronic kidney disorder as well as information on sexually transmitted diseases				

© 2023 Varghese JS et al. *JAMA Network Open.*
