# Supplementary material for: Hypertension Diagnosis, Treatment, and Control in India
Source: JAMA Netw Open. 2023 Oct 23;6(10):e2339098. doi: 10.1001/jamanetworkopen.2023.39098 (PMC10594142; doi:10.1001/jamanetworkopen.2023.39098)
Supplement: Supplement 2. — Data Sharing Statement [file jamanetwopen-e2339098-s002.pdf]

Data Sharing Statement

Varghese. Hypertension Diagnosis, Treatment, and Control in India. *JAMA Netw Open*. Published October 23, 2023. doi:10.1001/jamanetworkopen.2023.39098

Data
Data available: No

Additional Information
Explanation for why data not available: All datasets used in this analysis are available for download at www.dhsprogram.com. The code for the analysis is available on https://github.com/jvargh7/hypertension_cascade.
